# Co-circulation of SARS-CoV-2 Alpha and Gamma variants in Italy, February and March 2021

**DOI:** 10.2807/1560-7917.ES.2022.27.5.2100429

**Published:** 2022-02-03

**Authors:** Paola Stefanelli, Filippo Trentini, Giorgio Guzzetta, Valentina Marziano, Alessia Mammone, Monica Sane Schepisi, Piero Poletti, Carla Molina Grané, Mattia Manica, Martina del Manso, Xanthi Andrianou, Marco Ajelli, Giovanni Rezza, Silvio Brusaferro, Stefano Merler, COVID-19 National Microbiology Surveillance Study Group, COVID-19 National Microbiology Surveillance Study Group, Angela Di Martino, Luigina Ambrosio, Alessandra Lo Presti, Stefano Fiore, Concetta Fabiani, Eleonora Benedetti, Giuseppina Di Mario, Marzia Facchini, Simona Puzelli, Laura Calzoletti, Stefano Fontana, Giulietta Venturi, Claudia Fortuna, Giulia Marsili, Antonello Amendola, Liborio Stuppia, Giovanni Savini, Antonio Picerno, Teresa Lopizzo, Domenico Dell’Edera, Pasquale Minchella, Francesca Greco, Giuseppe Viglietto, Luigi Atripaldi, Antonio Limone, Pierlanfranco D’Agaro, Danilo Licastro, Stefano Pongolini, Vittorio Sambri, Giorgio Dirani, Paola Affanni, Maria Eugenia Colucci, Maria Rosaria Capobianchi, Giancarlo Icardi, Bianca Bruzzone, Flavia Lillo, Andrea Orsi, Elena Pariani, Fausto Baldanti, Maria Rita Gismondo, Fabrizio Maggi, Arnaldo Caruso, Ferruccio Ceriotti, Maria Beatrice Boniotti, Ilaria Barbieri, Patrizia Bagnarelli, Stefano Menzo, Silvio Garofalo, Massimiliano Scutellà, Elisabetta Pagani, Lucia Collini, Valeria Ghisetti, Silvia Brossa, Giuseppe Ru, Elena Bozzetta, Maria Chironna, Antonio Parisi, Salvatore Rubino, Caterina Serra, Giovanna Piras, Ferdinando Coghe, Francesco Vitale, Fabio Tramuto, Guido Scalia, Concetta Ilenia Palermo, Giuseppe Mancuso, Teresa Pollicino, Francesca Di Gaudio, Stefano Vullo, Stefano Reale, Maria Grazia Cusi, Gian Maria Rossolini, Mauro Pistello, Antonella Mencacci, Barbara Camilloni, Silvano Severini, Massimo Di Benedetto, Calogero Terregino, Isabella Monne, Valeria Biscaro

**Affiliations:** 1 Department of Infectious Diseases, Istituto Superiore di Sanità, Rome, Italy; 2 Center for Health Emergencies, Bruno Kessler Foundation, Trento, Italy; 3 Dondena Centre for Research on Social Dynamics and Public Policy, Bocconi University, Milan, Italy; 4 Directorate General of Health Prevention, Ministry of Health, Rome, Italy; 5 University of Trento, Trento, Italy; 6 European Programme for Intervention Epidemiology Training (EPIET), European Centre for Disease Prevention and Control (ECDC), Stockholm, Sweden; 7 Cyprus University of Technology, Limassol, Cyprus; 8 Laboratory for Computational Epidemiology and Public Health, Department of Epidemiology and Biostatistics, Indiana University School of Public Health, Bloomington, IN, United States; 9 Istituto Superiore di Sanità, Rome, Italy; 10 The members of the COVID-19 National Microbiology Surveillance Study Group are listed under Investigators and at the end of the article

**Keywords:** SARS-CoV-2 variant of concern, transmissibility, co-circulation, lineage

## Abstract

**Background:**

Several SARS-CoV-2 variants of concern (VOC) have emerged through 2020 and 2021. There is need for tools to estimate the relative transmissibility of emerging variants of SARS-CoV-2 with respect to circulating strains.

**Aim:**

We aimed to assess the prevalence of co-circulating VOC in Italy and estimate their relative transmissibility.

**Methods:**

We conducted two genomic surveillance surveys on 18 February and 18 March 2021 across the whole Italian territory covering 3,243 clinical samples and developed a mathematical model that describes the dynamics of co-circulating strains.

**Results:**

The Alpha variant was already dominant on 18 February in a majority of regions/autonomous provinces (national prevalence: 54%) and almost completely replaced historical lineages by 18 March (dominant across Italy, national prevalence: 86%). We found a substantial proportion of the Gamma variant on 18 February, almost exclusively in central Italy (prevalence: 19%), which remained similar on 18 March. Nationally, the mean relative transmissibility of Alpha ranged at 1.55–1.57 times the level of historical lineages (95% CrI: 1.45–1.66). The relative transmissibility of Gamma varied according to the assumed degree of cross-protection from infection with other lineages and ranged from 1.12 (95% CrI: 1.03–1.23) with complete immune evasion to 1.39 (95% CrI: 1.26–1.56) for complete cross-protection.

**Conclusion:**

We assessed the relative advantage of competing viral strains, using a mathematical model assuming different degrees of cross-protection. We found substantial co-circulation of Alpha and Gamma in Italy. Gamma was not able to outcompete Alpha, probably because of its lower transmissibility.

## Introduction

Since the end of 2020, multiple severe acute respiratory syndrome coronavirus 2 (SARS-CoV-2) variants of concern (VOC) have emerged across the globe. Some of them are particularly concerning as their biological characteristics allowed them to outcompete and rapidly replace historical lineages in the countries where they probably emerged, and to spread rapidly to many other countries. The Alpha variant (Phylogenetic Assignment of Named Global Outbreak (Pango) lineage designation: B.1.1.7) was first detected in the United Kingdom (UK) in samples from September 2020 and became dominant throughout the country by early 2021 [1,2]. It has spread in most of Europe and has been reported in a majority countries worldwide [3-6]. The Gamma variant (P.1) was first reported in Japan among travellers returning from Brazil [7]. It was later found in almost half of the cases in December 2020 in Manaus, Brazil where, despite a very high estimated seroprevalence against historical lineages, a large upsurge of infections occurred throughout January 2021 [8-10]. The Beta variant (B.1.351) was first detected in South Africa, where it became dominant in late November 2020 [11]. 

The epidemiological success of these variants relies on evolutionary advantages such as increased transmissibility [1,2,12] and their ability (demonstrated for Gamma and Beta) to significantly reduce antibody neutralisation in convalescent and post-vaccination sera [13-17], probably resulting in reinfections through immune escape [18-21]. Besides their greater ability to spread, requiring more restrictive physical distancing measures to mitigate epidemics, these variants have caused additional concern regarding potentially increased morbidity and mortality [22,23] as well as their potential impact on vaccine effectiveness [24,25]. Because variants seem to have emerged in unconnected geographical areas, little is known as yet about their ecological interactions. 

Here, we have developed a three-strain susceptible–infectious–recovered (SIR) model that simulates the co-circulation of historical SARS-CoV-2 lineages (wildtype) and the Alpha and Gamma VOC under different assumptions regarding the degree of cross-protection, to estimate the relative transmissibility of emerging variants vs historical lineages, using data from genomic surveillance in Italy. This model offers a simple and efficient tool to estimate the relative transmissibility of newly emerging variant of the SARS-CoV-2 virus such as the Delta variant (B.1.617.2) with respect to circulating variants.

## Methods

### Epidemiological information

Publicly available data on the number of newly diagnosed SARS-CoV-2 infections were taken from the Italian Civil Protection Department [26]. Data on the number of newly admitted patients in hospitals were obtained from the Italian integrated surveillance system curated by the Italian National Institute of Health (Istituto Superiore di Sanità, ISS) [27,28]. Data on physical distancing restrictions were collected from the Italian Ministry of Health [29]. Physical distancing restrictions were organised in tiers of different colours (white, yellow, orange and red, from most relaxed to most strict) [30] and updated for each Italian region on a weekly basis (with occasional exceptions). Restrictions corresponding to each tier are fully described in the Supplement. For purely descriptive reasons, we computed a tier index summarising the stringency of restrictions across the national territory: tiers were assigned an arbitrary value from 0 (white) to 3 (red) and their average weighted by the proportion of the population living under each tier was computed for each day. The resulting value was normalised to have values between 0 (representing 100.0% of the population under the white tier) and 1 (100.0% of the population under the red tier).

### Survey methodology

Two cross-sectional surveys coordinated by the Italian National Institute of Health, in collaboration with the Ministry of Health and the laboratories of regions/autonomous provinces (AP) were conducted on 18 February and 18 March 2021, to estimate the prevalence of the Alpha, Gamma, and Beta variants [31,32].

The surveys involved all 19 regions and the two AP of Italy. Random samples of SARS-CoV-2-positive cases, diagnosed on 18 February and 18 March with a real-time reverse transcription PCR (RT-PCR) and a quantification cycle (Cq) < 28, were analysed in 101 and 129 laboratories, respectively, distributed across the national territory. Samples came from both passive surveillance and contact tracing and included symptomatic, pre-symptomatic and asymptomatic COVID-19 cases.

Samples were distributed across five macro-areas, defined according to the Eurostat NUTS1 classification: North-east, North-west, Centre, South and Islands. The sample size was calculated to have the statistical power to detect a prevalence of 1%, with 0.8% error within each macro-area, based on the number of cases notified on the day preceding each of the two surveys [33]. The collected samples were sequenced according to the local laboratory policy by either of the following techniques: (i) sequencing the entire S gene by Sanger technology, (ii) sequencing part of the S gene with identification of all mutations/deletions associated with SARS-CoV-2 variants or (iii) sequencing the whole genome by next generation sequencing. A small fraction of the sequenced samples could not be analysed because of insufficient sequencing coverage of the genome and were therefore discarded. In the second survey, one region (Marche) pre-screened 54 of its 65 RT-PCR-positive samples using an in-house test (see the Supplement for the methodology) that detects both H69-V70 and Y144 amino acid deletions which are specific for the Alpha variant; as a result, 46 cases positive in the in-house test were considered to be Alpha without sequencing. The eight cases negative in the test, plus the 11 cases that were not pre-screened were sequenced.

The point prevalence of the three lineages in each survey was computed as the fraction of infections confirmed for each lineage among sequenced samples, and corresponding multinomial 95% confidence intervals (CI) are provided. Multinomial CI were computed using the function MultinomlCI with Wilson method from package DescTools in R version 3.6.2. In the second survey, data from Marche were excluded from all analyses.

### SARS-CoV-2 three-strain transmission model

We adopted a three-strain SIR mathematical model to simulate co-circulation of historical lineages of SARS-CoV-2 (wildtype) and the VOC Alpha and Gamma. We did not consider the Beta variant because the surveys found little or no circulation of this lineage (see Results). We assumed that a previous infection with the wildtype provides complete protection against the Alpha variant [34] and that infection with either the wildtype or Alpha confers the same degree of cross-protection against the Gamma variant. In addition, we assumed that the transmissibility of variants Alpha and Gamma are scaled with respect to the wildtype transmissibility by a lineage-specific factor representing their relative transmissibility. We assumed that the three strains had identical generation time (set at 6.6 days to reflect the serial interval estimated for SARS-CoV-2 in Italy [35]). The model was initialised on 15 January 2021, assuming that an unknown fraction of all infections on that date belonged to the Alpha and Gamma variant (see the Supplement for full model details). Unknown model parameters (namely, the transmissibility of the wildtype strain, the relative transmissibility parameters of Alpha and Gamma and their respective initial prevalence) were estimated by calibrating the model against prevalence data from the two surveys, using a Markov chain Monte Carlo (MCMC) approach based on Metropolis–Hastings sampling and uninformative priors. Different scenarios for cross-protection from wildtype and Alpha against Gamma were explored. The likelihood was defined as:


$$L=\prod_{t}M(S_{t\;}^{wildtype},{S_{t\;}^{Alpha},\;S_{t}^{Gamma},Q}_{t},p_{t}^{wildtype},\;p_{t}^{Alpha},\;p_{t}^{Gamma}\;)$$

Where *M(·)* is the multinomial probability density distribution, t is the date of the survey, *S^l^_t_* is the number of cases observed at date *t* for lineage *l*, *Q_t_ = ∑_l_S^l^_t_* is the total number of analysed samples at date *t*, and *p^l^_t_* is the model-estimated fraction of infections of lineage *l* over the total at date *t*. In order to reproduce the observed epidemiological temporal trends, we assigned *L =* 0 to simulations for which the model’s mean squared error on the observed daily hospital admissions between 18 January and 18 March 2021 exceeded 1.5 times the variance of observations. We ran the MCMC algorithm for 50,000 iterations and convergence was assessed by checking that, after a burn-in period of 10,000 iterations, the trace plots associated with different chains, i.e. the sequence of accepted parameter values, were characterised by an approximately constant standard deviation and average, therefore proving good mixing of the parameters.

The model was run on four different geographical aggregations of regional data, i.e. the national level and the Centre, North-east and South macro-areas. Macro-areas North-west and Islands did not have samples positive for the Gamma variant. Data from Marche were excluded because of heterogeneity in data collection, but we performed a sensitivity analysis to evaluate the impact of this choice. We also ran additional sensitivity analyses to evaluate the robustness of results (see the Supplement for the results of these sensitivity analyses). Specifically, we estimated the relative transmissibility of Alpha alone in Lombardy and Veneto, where Gamma was not co-circulating and for which a previous data point on the prevalence of Alpha was available from a survey conducted with the same methodology on cases confirmed on 3–4 February (the relative transmissibility of Alpha in this scenario is provided in the Supplement). To evaluate the potential impact of geographical aggregation on estimates of the relative transmissibility of Alpha and Gamma, we re-ran the analysis in two individual regions with high co-circulation (Tuscany and Lazio). Finally, we ran an analysis where, instead of fixing the degree of cross-protection between historical, Alpha and Gamma lineages, we left it as a free model parameter. 

Estimated posterior distributions of free parameters are summarised by posterior means and 95% credible intervals (CrI). 

## Ethical statement

Ethical approval for sequencing SARS-CoV-2 genomes from clinical samples sent to ISS was obtained from the Ethical Committee of ISS (ref. PRE BIO CE n.26259, 29 July 2020).

## Results

### SARS-CoV-2 epidemiology in Italy, January–March 2021

**Figure 1:**
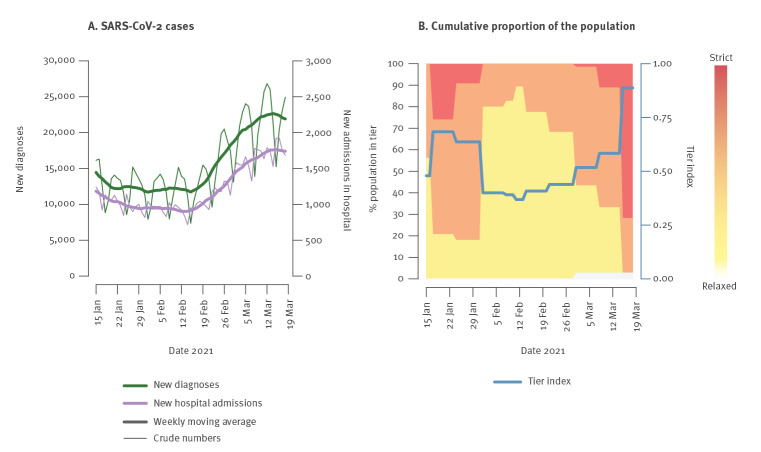
Epidemiological COVID-19 situation in Italy between 15 January and 18 March 2021

The daily incidence of newly diagnosed SARS-CoV-2 cases and COVID-19 patients admitted to hospital remained stable in Italy between mid-January and mid-February 2021, then increased in the second half of February and first half of March (Figure 1A) [26]. Strict measures in the second half of January (ca 80% of the population in orange/red tiers, Figure 1B) were relaxed in February and remained similar until March (ca 70–80% of citizens in yellow tier, none in red tier). Starting on 14 March, restrictions were rapidly tightened to control the rise in infections and hospitalisations, and more than 70% of the national population ended up in the strictest tier.

COVID-19: coronavirus disease.

Data sources for panel A: new diagnoses [26]; hospital admissions [27]. Data source for panel B: population in white, yellow, orange and red tiers [29]. A value of 0 for the tier index implies that the entire population is in the most relaxed tier (white) and a value of 1 implies that the entire population is in the most strict tier (red).

### Prevalence of variants of concern in Italy, 18 February and 18 March

**Figure 2:**
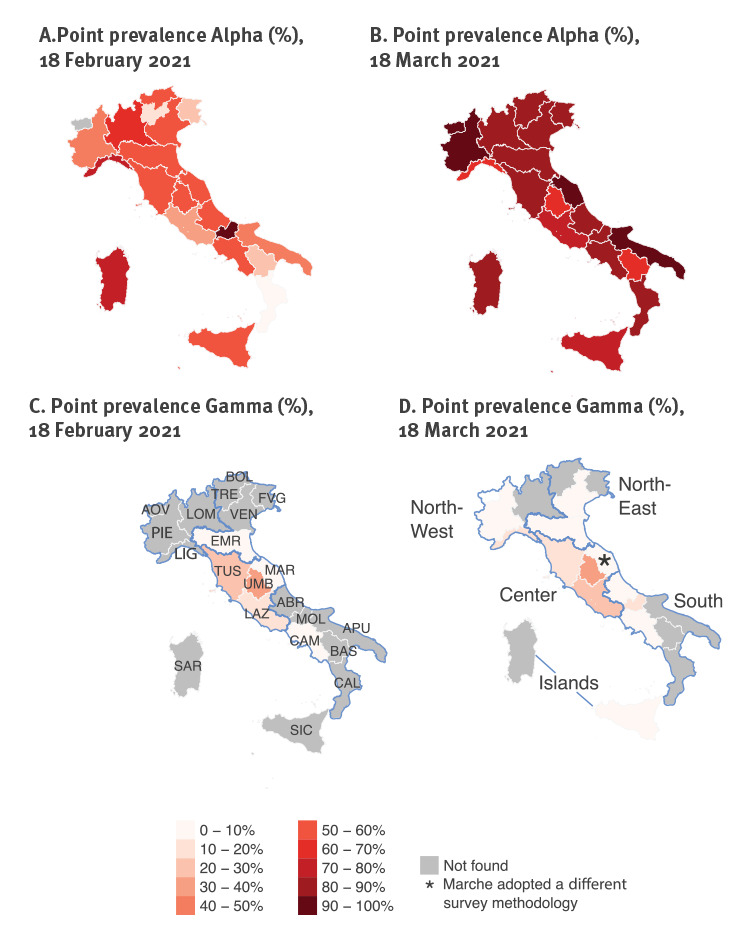
Geographical distribution of SARS-CoV-2 variants of concern, Italy, point prevalence on 18 February 2021 (n = 1,296) and 18 March 2021 (n = 1,938)

During the genomic survey conducted on 18 February, 1,296 samples were sequenced, of which 57 (4%) were discarded for the analysis because of insufficient sequencing coverage of the genome. Among the remaining 1,239 analysed samples, the Alpha variant was confirmed in 658, Gamma in 62 and Beta in six, for a national prevalence of 53.1% (95% CI: 50.2–56.1), 5.0% (95% CI: 2.1–7.9) and 0.5% (95% CI: 0.0–3.4), respectively. The Alpha variant was found in 20 of 21 regions/AP, Gamma in six and Beta in three (Figure 2 and Table 1). The prevalence of Alpha was highest in the North-west macro-area (60.4%; 95% CI: 55.1–66.1) and lowest in the Centre (44.7%; 95% CI: 38.8–50.8), while Gamma was almost exclusively concentrated in the Centre (mean prevalence: 18.8%; 95% CI: 12.9–24.9, as opposed to 1% or less elsewhere; see Table 1 and Figure 2). The Beta variant was identified only in Lombardy (three cases), in the AP Bolzano (two cases) and in Sicily (one case).

SARS-CoV-2: severe acute respiratory syndrome coronavirus 2.

Abbreviations of region names are reported in panel C and listed in the Supplement. Borders of mainland macro-areas are highlighted in blue, and the names of macro-areas are reported in panel D.

**Table 1:** Results of the first SARS-CoV-2 point prevalence survey across the 21 participating regions/AP, Italy, 18 February 2021 (n = 1,296)

Region	Laboratories	RT-PCR-positive	Sequenced samples	Analysed samples	Confirmed cases			Point prevalence (95% CI)		
					Alpha	Gamma	Beta	Alpha	Gamma	Beta
Islands										
Sardinia	6	38	25	12	9	0	0	75% (46.8–91.1)	0% (0–24.2)	0% (0–24.2)
Sicily	5	268	63	58	32	0	1	55.2% (42.5–67.3)	0% (0–6.2)	1.7% (0.3–9.1)
**Total islands**	**11**	**306**	**88**	**70**	**41**	**0**	**1**	**58.6% (46.9–69.4)**	**0% (0–5.2)**	**1.4% (0.3–7.7)**
South										
Abruzzo	2	374	61	61	31	0	0	50.8% (38.6–62.9)	0% (0–5.9)	0% (0–5.9)
Apulia	7	59	59	59	28	0	0	47.5% (35.3–60)	0% (0–6.1)	0% (0–6.1)
Basilicata	5	7	7	5	1	0	0	20% (3.6–62.4)	0% (0–43.4)	0% (0–43.4)
Calabria	3	166	11	11	1	0	0	9.1% (1.6–37.7)	0% (0–25.9)	0% (0–25.9)
Campania	2	366	86	86	51	2	0	59.3% (48.7–69.1)	2.3% (0.6–8.1)	0% (0–4.3)
Molise	1	114	15	15	14	0	0	93.3% (70.2–98.8)	0% (0–20.4)	0% (0–20.4)
**Total South**	**20**	**1,086**	**239**	**237**	**126**	**2**	**0**	**53.2% (46.8–59.4)**	**0.8% (0.2–3)**	**0% (0–1.6)**
Centre										
Lazio	5	169	169	144	49	19	0	34% (26.8–42.1)	13.2% (8.6–19.7)	0% (0–2.6)
Marche	8	38	38	38	22	3	0	57.9% (42.2–72.1)	7.9% (2.7–20.8)	0% (0–9.2)
Tuscany	3	88	80	80	43	19	0	53.8% (42.9–64.3)	23.8% (15.8–34.1)	0% (0–4.6)
Umbria	4	247	48	47	24	17	0	51.1% (37.2–64.7)	36.2% (24–50.5)	0% (0–7.6)
**Total Centre**	**20**	**542**	**335**	**309**	**138**	**58**	**0**	**44.7% (39.2–50.2)**	**18.8% (14.8–23.5)**	**0% (0–1.2)**
North-east										
AP Bolzano	1	320	70	70	40	0	2	57.1% (45.5–68.1)	0% (0–5.2)	2.9% (0.8–9.8)
AP Trento	1	20	20	14	2	0	0	14.3% (4–39.9)	0% (0–21.5)	0% (0–21.5)
Emilia-Romagna	2	99	99	99	57	2	0	57.6% (47.7–66.8)	2% (0.6–7.1)	0% (0–3.7)
Friuli Venezia Giulia	4	133	28	27	8	0	0	29.6% (15.9–48.5)	0% (0–12.5)	0% (0–12.5)
Veneto	12	92	92	92	52	0	0	56.5% (46.3–66.2)	0% (0–4)	0% (0–4)
**Total North-east**	**20**	**664**	**309**	**302**	**159**	**2**	**2**	**52.6% (47–58.2)**	**0.7% (0.2–2.4)**	**0.7% (0.2–2.4)**
North-west										
Aosta Valley	1	1	1	1	0	0	0	0% (0–79.3)	0% (0–79.3)	0% (0–79.3)
Liguria	6	227	22	22	16	0	0	72.7% (51.8–86.8)	0% (0–14.9)	0% (0–14.9)
Lombardy	9	213	213	213	137	0	3	64.3% (57.7–70.4)	0% (0–1.8)	1.4% (0.5–4.1)
Piedmont	14	93	89	85	41	0	0	48.2% (37.9–58.7)	0% (0–4.3)	0% (0–4.3)
**Total North-west**	**30**	**534**	**325**	**321**	**194**	**0**	**3**	**60.4% (55–65.6)**	**0% (0–1.2)**	**0.9% (0.3–2.7)**
Total Italy	101	3,132	1,296	1,239	658	62	6	53.1% (50.3–55.9)	5% (3.9–6.4)	0.5% (0.2–1.1)

CI: confidence interval; SARS-CoV-2: severe acute respiratory syndrome coronavirus 2.

All percentages in the table are approximated to the first decimal, the value zero may therefore stand for < 0.05%. ‘RT-PCR-positive’ represent only cases tested in the laboratories involved and not all positive cases in an area.

During the genomic survey conducted on 18 March, 1,938 samples were sequenced (not including data from Marche), of which 24 (1%) were discarded for the analysis because of insufficient coverage of the genome sequencing (Table 2). Among the remaining 1,914 analysed samples, Alpha was confirmed in 1,641 infections, Gamma in 92 and Beta in three, for a national prevalence of 85.7% (95% CI: 84.3–87.3), 4.8% (95% CI: 3.3–6.3) and 0.2% (95% CI: 0.0–1.7), respectively. Alpha was found in all 21 regions/AP, Gamma in 12, and Beta in three (Figure 2 and Table 2). According to the survey conducted on 18 March, the Alpha variant had become dominant in all Italian regions, with regional prevalence estimates ranging from 63.6% to 100.0% (Figure 2 and Table 2). Regional prevalence estimates for the Gamma variant ranged from 0.0% to 32%; the highest prevalence estimates for this VOC were still obtained for central regions (Figure 2), however, it was detected in six additional regions compared with 18 February. The Beta variant was identified only in Lombardy, Sardinia and Veneto (one case each).

**Table 2:** Results from the second SARS-CoV-2 point prevalence survey across the 21 participating regions/AP, Italy, 18 March 2021 (n = 1,938)

Region	Laboratories	RT-PCR-positive	Sequenced samples	Analysed samples	Confirmed cases			Point prevalence (95% CI)		
					Alpha	Gamma	Beta	Alpha	Gamma	Beta
Islands										
Sardinia	6	85	21	21	18	0	1	85.7% (65.4–95)	0% (0–15.5)	4.8% (0.8–22.7)
Sicily	5	632	132	129	97	3	0	75.2% (67.1–81.8)	2.3% (0.8–6.6)	0% (0–2.9)
**Total Islands**	**11**	**717**	**153**	**150**	**115**	**3**	**1**	**76.7% (69.3–82.7)**	**2% (0.7–5.7)**	**0.7% (0.1–3.7)**
South										
Abruzzo	2	293	87	80	66	4	0	82.5% (72.7–89.3)	5% (2–12.2)	0% (0–4.6)
Apulia	11	126	126	126	117	0	0	92.9% (87–96.2)	0% (0–3)	0% (0–3)
Basilicata	6	62	27	20	13	0	0	65% (43.3–81.9)	0% (0–16.1)	0% (0–16.1)
Calabria	4	404	26	26	22	0	0	84.6% (66.5–93.8)	0% (0–12.9)	0% (0–12.9)
Campania	3	1400	261	261	232	4	0	88.9% (84.5–92.2)	1.5% (0.6–3.9)	0% (0–1.5)
Molise	1	63	16	16	13	2	0	81.2% (57–93.4)	12.5% (3.5–36)	0% (0–19.4)
**Total South**	**27**	**2,348**	**543**	**529**	**463**	**10**	**0**	**87.5% (84.4–90.1)**	**1.9% (1–3.4)**	**0% (0–0.7)**
Centre										
Lazio	11	214	205	205	161	42	0	78.5% (72.4–83.6)	20.5% (15.5–26.5)	0% (0–1.8)
Marche^a^	11	65	Excluded from analysis^a^					83.1% (72.2–90.3)	3.1% (0.8–10.5)	0% (0–5.6)
Tuscany	3	144	103	99	85	10	0	85.9% (77.7–91.4)	10.1% (5.6–17.6)	0% (0–3.7)
Umbria	5	80	26	25	16	8	0	64% (44.5–79.8)	32% (17.2–51.6)	0% (0–13.3)
**Total Centre^b^**	**30**	**503**	**334**	**329**	**262**	**60**	**0**	**79.6% (75–83.6)**	**18.2% (14.4–22.8)**	**0% (0–1.2)**
North-east										
Ap Bolzano	1	69	15	15	12	0	0	80% (54.8–93)	0% (0–20.4)	0% (0–20.4)
Ap Trento	1	16	16	16	16	0	0	100% (80.6–100)	0% (0–19.4)	0% (0–19.4)
Emilia-Romagna	2	175	175	175	154	13	0	88% (82.4–92)	7.4% (4.4–12.3)	0% (0–2.1)
Friuli Venezia Giulia	7	126	55	55	49	0	0	89.1% (78.2–94.9)	0% (0–6.5)	0% (0–6.5)
Veneto	13	156	156	156	138	2	1	88.5% (82.5–92.6)	1.3% (0.4–4.6)	0.6% (0.1–3.5)
**Total North-east**	**24**	**542**	**417**	**417**	**369**	**15**	**1**	**88.5% (85.1–91.2)**	**3.6% (2.2–5.8)**	**0.2% (0–1.3)**
North-west										
Aosta Valley	1	32	2	2	2	0	0	100% (34.2–100)	0% (0–65.8)	0% (0–65.8)
Liguria	8	179	22	22	14	3	0	63.6% (43–80.3)	13.6% (4.7–33.3)	0% (0–14.9)
Lombardy	12	314	314	312	278	0	1	89.1% (85.2–92.1)	0% (0–1.2)	0.3% (0.1–1.8)
Piedmont	16	155	153	153	138	1	0	90.2% (84.5–94)	0.7% (0.1–3.6)	0% (0–2.4)
**Total North-west**	**37**	**680**	**491**	**489**	**432**	**4**	**1**	**88.3% (85.2–90.9)**	**0.8% (0.3–2.1)**	**0.2% (0–1.1)**
Total Italy^b^	129	4,790	1,938	1,914	1,641	92	3	85.7% (84.1–87.2)	4.8% (3.9–5.9)	0.2% (0.1–0.5)

CI: confidence interval; SARS-CoV-2: severe acute respiratory syndrome coronavirus 2.

All percentages in the table are approximated to the first decimal, the value zero may therefore stand for < 0.05%. ‘RT-PCR-positive’ represent only cases tested in the laboratories involved and not all positive cases in an area.

^a^ Marche followed a different experimental design (see Methods).

**^b^** The prevalence and totals for Italy and macro-area Centre do not include results from Marche.

### Relative transmissibility of SARS-CoV-2 variants Alpha and Gamma

**Figure 3:**
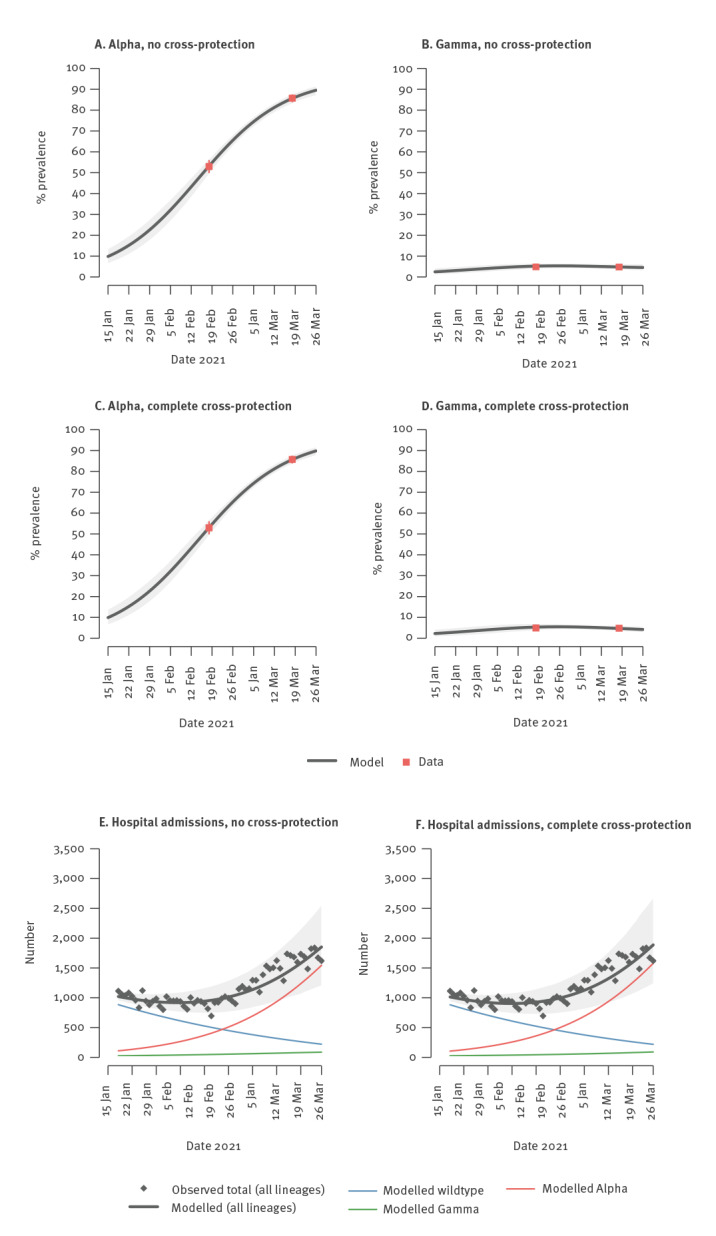
Model fits for prevalence of SARS-CoV-2 variants of concern and hospital admissions, Italy, 18 January–18 March 2021

CrI: credible intervals; SARS-CoV-2: severe acute respiratory syndrome coronavirus 2.

The three-strain transmission model was able to fit the epidemiological trends on hospital admissions and the estimated prevalence of the Alpha and Gamma VOC in all geographical aggregations and independently of the assumed degree of cross-protection (Figure 3 shows results for Italy when assuming no cross-protection or complete cross-protection).

Panels A–D: Model estimates, solid black lines: mean values; shaded areas: 95% CrI. Observed data, red points: mean values: red lines: 95% CrI. At each time, the model-estimated prevalence for a given lineage is computed as the ratio of simulated number of infectious individuals with that lineage and the simulated total number of infectious individuals. Panels E and F: Model-estimated and observed hospital admissions over time under the assumption of no cross-protection between wildtype or Alpha and Gamma. Black lines: mean values of model-estimated overall daily hospitalisations; shaded areas: 95%CrI. Coloured lines: mean values of model-estimated daily hospitalisations; black points: observed daily hospitalisations

**Figure 4:**
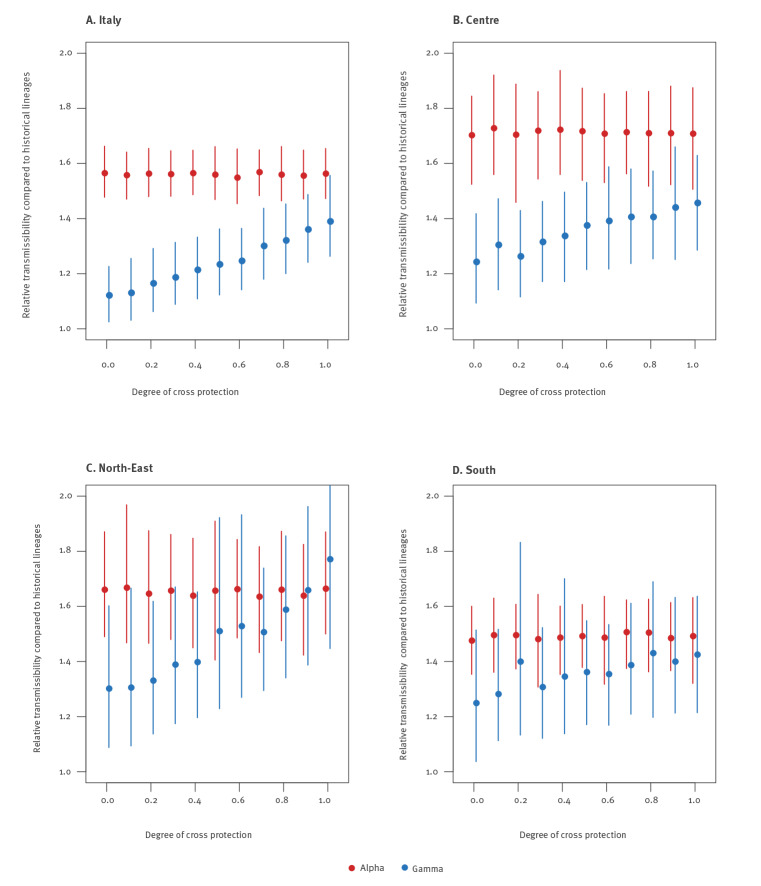
Estimates of the relative transmissibility of SARS-CoV-2 variants Alpha and Gamma, Italy, 18 February and 18 March 2021

Independently of the geographical aggregation and of the assumed degree of cross-protection, we found a robust mean estimate for the relative transmissibility of Alpha, ranging between 1.48 and 1.73, with CrI ranging between 1.31 and 1.97 (Figure 4). Considering the national aggregation, estimates varied between 1.55 and 1.57 with CrI ranging between 1.45 and 1.66. When estimating the relative transmissibility of Alpha in the absence of significant co-circulation of Gamma, using data from Veneto and Lombardy, we found consistent values of 1.49 (95% CrI: 1.36–1.66) and 1.72 (95% CrI: 1.52–1.98) respectively (see the Supplement for results in this scenario).

SARS-CoV-2: severe acute respiratory syndrome coronavirus 2.

Estimates are provided for different assumed values on the degree of cross protection (0: no cross-protection; 1: complete cross-protection) conferred by previous infection with the wildtype or Alpha against reinfection with Gamma. Points indicate the mean value of the estimated relative transmissibility; lines indicate 95% credible intervals. The panels represent four geographical aggregations for which the analysis was possible given the presence of the Gamma. Data from Marche were excluded from the national estimate and the macro-area Centre because of heterogeneity in data collection.

The estimated relative transmissibility of the Gamma variant was systematically lower than the one estimated for Alpha. For the national aggregation and for the three regions of the macro-area Centre (Lazio, Tuscany and Umbria) where the observed prevalence of Gamma was higher, we estimate a relative transmissibility of 1.12–1.24 under the assumption that there is no cross-protection (95% CrI: 1.03–1.42), growing linearly for increasing values of cross-protection, up to 1.39–1.46 (95% CrI: 1.26–1.63) under the assumption of complete cross-protection. Results for the macro-areas North-east and South reproduced a similar pattern, although these estimates were more variable because of the limited number of reported Gamma. The results remained robust when considering individual regions with high co-circulation, and when the degree of cross-protection was not assumed a priori but considered as a free model parameter (see the Supplement for detailed results of this sensitivity analysis).

## Discussion

We provide a mathematical framework to estimate the relative transmissibility of competing SARS-CoV-2 variants of concern, taking as case study the co-circulation of Alpha and Gamma in Italy at the beginning of 2021. Based on two genomic surveillance surveys conducted across the whole Italian territory on 18 February and 18 March 2021, we showed that the Alpha variant was already dominant on 18 February in a majority of regions/AP (national prevalence: 54%) and had almost completely replaced historical lineages by 18 March (dominant in all regions/AP, national prevalence: 86%). At the same time, we found a substantial proportion of cases of the Gamma variant on 18 February, almost exclusively in Regions of central Italy (Lazio, Tuscany, Umbria and Marche, with an overall prevalence of 19%). Gamma was also identified in samples from Campania and Emilia Romagna, both with prevalences below 3%. The prevalence of Gamma remained similar on 18 March, suggesting that this VOC was not able to outcompete Alpha. However, on 18 March, lineage Gamma was identified in cases from six additional regions in northern (Piedmont, Veneto, Liguria) and southern Italy (Abruzzo, Molise, Sicily). We found only six cases of the Beta variant among the 1,239 analysed samples on 18 February, and only three of 1,908 on 18 March. Using data from these two surveys, we made use of a mathematical transmission model to estimate the relative advantage of these two VOC over the wildtype virus. Compared with historical lineages, we estimated a mean relative transmissibility of Alpha ranging between 1.55 and 1.57 (with 95% CrI between 1.45 and 1.66) in Italy. These values are consistent with available estimates from the UK [1,2,36,37] and France [12].

The estimated relative transmissibility of Gamma (compared with historical lineages) varied according to different assumptions on the degree of cross-protection granted by previous infection with historical lineages or Alpha: the estimate at the national level ranged from 1.12 (95% CrI: 1.03–1.23) in the case of complete immune evasion by Gamma to 1.39 (95% CrI: 1.26–1.56) in the case of complete cross-protection. This transmissibility advantage estimated for Gamma would have been sufficient for replacement of historical lineages in the absence of Alpha, independently of the degree of cross-protection (see the Supplement for detailed additional results). Previous estimates on the relative transmissibility of Gamma provided from a study in Manaus, Brazil, where the variant rapidly replaced historical lineages, were very broad (between 1.03 and 2.87), with an estimate of cross-protection between 12% and 90.0%.

As suggested by our work, the true degree of cross-protection between variants of concern is likely to be critical for their coexistence, and a key role will be played by the effectiveness of licensed vaccines against emerging strains. The slight decrease in Gamma prevalence over 1 month occurred under a condition of strict mitigation measures; if Gamma could have at least partially escaped immunity from infection with Alpha and from existing vaccines, this may have posed challenges to the roll-out of vaccination programmes. Furthermore, if some degree of cross-protection exists, a higher proportion of asymptomatic infections may have been observed, posing challenges to surveillance and control. It is crucial to keep continuously monitoring the relative advantage of newly emerging VOC to efficiently contain or mitigate outbreaks and evaluate the impact of the adopted interventions.

We acknowledge a number of limitations for this study. The sample size was calculated to have the statistical power to detect different lineages at the macro-area level. As such, regional estimates of prevalence should be taken with caution because the number of sequenced samples was small. Samples were randomly selected for sequencing among cases diagnosed by the laboratories, but some degree of correlation between them (e.g. cases belonging to an over-represented cluster on that day) cannot be completely excluded, especially in regions with smaller sample sizes. One region used a different laboratory methodology in the second survey and those data were excluded from the computation of the national prevalence of the variants. Possible biases in the estimate are expected to be minimal, since cases from this region represented only ca 1.5% of the total. The model was calibrated using available data on two genomic surveys only, and this represents a limitation of our study. However, for biological reasons, we assumed a simple exponential growth model for the estimation of the prevalence of Alpha and Gamma variants, according to which the prevalence over time is a logistic function, and this function is univocally defined by two points in time. As future measurements of the prevalence of co-circulating variants become available, a validation of the estimated prevalence over time is warranted.

Regarding model estimates on transmissibility, we could not take into account major determinants of SARS-CoV-2 transmission dynamics such as possible differences across strains in the generation time and duration of viral shedding [38], age-specific susceptibility or transmissibility [39-41], the age-specific proportion of asymptomatic individuals and their relative transmissibility [39], the severity of symptoms [42,43], because this kind of data on the variants was not available. For example, we show that if the duration of infectiousness, rather than the transmissibility, were responsible for the competitive advantage of Alpha over the historical lineages, we would expect a much higher increase in the duration of infection (80–150%) compared with the estimated increase in transmissibility (see the Supplement for detailed results of this sensitivity analysis). This would have implications for the preventive effectiveness of timely isolation of individuals and for quarantine times. Owing to these uncertainties, it is virtually impossible at this stage to model the potential lineage-specific impact of existing mitigation measures, nor to factor in the potential effect of vaccines administered until now. These factors are likely to shape the future outcome of the epidemiological competition across strains. For these reasons, we acknowledge that this work represents only a first step towards the understanding of the relationship among multiple SARS-CoV-2 variants and that our findings need to be corroborated by further evidence taking into account more details on the characteristics of emerging lineages as they become consolidated. Finally, our model did not consider possible differences in the interventions in place at regional level, and this may have resulted in heterogeneous estimates of the relative transmissibility of the variants in different areas of interest.

Despite these limitations, we provide a simple and useful modelling framework to assess the relative advantage of competing strains, by assuming different degrees of cross-protection among them. We found that the Gamma variant was not able to outcompete Alpha in Italy under existing mitigation measures, with Alpha becoming largely dominant and Gamma remaining stable over a month of co-circulation. We suggest that this may be due to a lower transmissibility of Gamma compared with Alpha and independent of its ability to re-infect individuals with previous infections with historical strains or the Alpha variant.

## Conclusion

We believe that our work provides an important tool to evaluate the relative transmissibility advantage of the SARS-CoV-2 Delta variant and of other newly emerging strains in the foreseeable future.

## COVID-19 National Microbiology Surveillance Study Group

Angela Di Martino, Luigina Ambrosio, Alessandra Lo Presti, Stefano Fiore, Concetta Fabiani, Eleonora Benedetti, Giuseppina Di Mario, Marzia Facchini, Simona Puzelli, Laura Calzoletti, Stefano Fontana, Giulietta Venturi, Claudia Fortuna, Giulia Marsili, Antonello Amendola, Department of Infectious Diseases, Istituto Superiore di Sanità, Rome; Liborio Stuppia, Laboratorio di Genetica Molecolare, Center for Advanced Studies and Technology (CAST), Università degli Studi “G. d'Annunzio”, Chieti; Giovanni Savini, Istituto Zooprofilattico Sperimentale dell'Abruzzo e del Molise “Giuseppe Caporale”, Teramo; Antonio Picerno, Teresa Lopizzo, UOC Analisi Chimico Cliniche e Microbiologiche, AOR “San Carlo”, Potenza; Domenico Dell’Edera, UOSD Laboratorio di Genetica Medica, P.O. “Madonna delle Grazie”, Matera; Pasquale Minchella, SOC Microbiologia e Virologia, AO “Pugliese-Ciaccio”, Catanzaro; Francesca Greco, UOC Microbiologia e Virologia, PO “Annunziata”, Cosenza; Giuseppe Viglietto, Laboratorio di Genomica Funzionale e Patologia Molecolare, Università degli Studi “Magna Graecia”, Catanzaro; Luigi Atripaldi, AORN “Azienda Sanitaria dei Colli”, Napoli; Antonio Limone, Istituto Zooprofilattico Sperimentale del Mezzogiorno, Napoli; Pierlanfranco D’Agaro, SC UCO Igiene e Sanità Pubblica, Azienda Sanitaria Universitaria Giuliano-Isontina (ASUGI), Trieste; Danilo Licastro, Laboratorio Genomica ed Epigenomica, Area Science Park, Basovizza, Trieste; Stefano Pongolini, Unità di Analisi del Rischio ed Epidemiologia Genomica, Istituto Zooprofilattico Sperimentale della Lombardia e dell’Emilia-Romagna, Parma; Vittorio Sambri, Dipartimento di Medicina Specialistica Diagnostica e Sperimentale (DIMES), Università di Bologna, Bologna, & UOC Microbiologia, AUSL della Romagna, Cesena; Giorgio Dirani, Silvia Zannoli, UOC Microbiologia, AUSL della Romagna, Cesena; Paola Affanni, Maria Eugenia Colucci, Laboratorio di Igiene e Sanità Pubblica, Dipartimento di Medicina e Chirurgia, Università degli Studi di Parma, Parma; Maria Rosaria Capobianchi, Laboratorio di Virologia, Istituto Nazionale Malattie Infettive IRCCS “L. Spallanzani”, Roma; Giancarlo Icardi, Bianca Bruzzone, Flavia Lillo, Andrea Orsi, Laboratorio di Riferimento Regionale per le Emergenze di Sanità Pubblica (LaRESP), Liguria; Elena Pariani, Dipartimento di Scienze Biomediche per la Salute, Università degli Studi di Milano, Milano; Fausto Baldanti, Unità Virologia Molecolare, Fondazione IRCCS Policlinico “San Matteo”, Università di Pavia, Pavia; Maria Rita Gismondo, UOC Microbiologia Clinica, Virologia e diagnostica delle Bioemergenze, ASST “Fatebenefratelli-Sacco”, Milano; Fabrizio Maggi, SC Laboratorio Microbiologia, ASST “Sette Laghi”, Varese; Arnaldo Caruso, Laboratorio di Microbiologia e Virologia, ASST “Spedali Civili di Brescia”, Brescia; Ferruccio Ceriotti, Fondazione IRCCS “Ca' Granda” Ospedale Maggiore Policlinico di Milano, Milano; Maria Beatrice Boniotti, Ilaria Barbieri, Istituto Zooprofilattico Sperimentale della Lombardia e dell'Emilia Romagna, Brescia; Patrizia Bagnarelli, Stefano Menzo, AOU “Ospedali Riuniti”, Ancona; Silvio Garofalo, Massimiliano Scutellà, Ospedale “Cardarelli”, Campobasso; Elisabetta Pagani, Laboratorio Aziendale di Microbiologia e Virologia, Azienda Sanitaria dell’Alto Adige, Bolzano; Lucia Collini, Microbiologia e Virologia, Presidio Ospedaliero “Santa Chiara”, Trento; Valeria Ghisetti, Laboratorio di Microbiologia e Virologia, ASL Città di Torino, Torino; Silvia Brossa, IRCCS Fondazione del Piemonte per l'Oncologia, Candiolo, Torino; Giuseppe Ru, Elena Bozzetta, Istituto Zooprofilattico Sperimentale del Piemonte, Liguria e Valle d’Aosta, Torino; Maria Chironna, Laboratorio di Epidemiologia Molecolare e Sanità Pubblica, AOUC Policlinico di Bari, Bari; Antonio Parisi, Istituto Zooprofilattico Sperimentale della Puglia e della Basilicata, Putignano, Bari; Salvatore Rubino, Caterina Serra, Department of Biomedical Science, University of Sassari, SC Microbiologia e Virologia, AOU Sassari, Sassari; Giovanna Piras, UOC Ematologia, PO “San Francesco,” Azienda Tutela Salute, ASSL Nuoro, Nuoro; Ferdinando Coghe, Laboratorio Generale (HUB) di analisi chimico cliniche e microbiologia, PO “Duilio Casula”, AOU di Cagliari, Cagliari; Francesco Vitale, Fabio Tramuto, Laboratorio di Riferimento Regionale per la Sorveglianza Epidemiologica e Virologica del PROMISE - AOUP “Giaccone”, Palermo; Guido Scalia, Concetta Ilenia Palermo, Laboratorio di Virologia Clinica, AOUP “V. Emanuele”, PO “Gaspare Rodolico”, Catania; Giuseppe Mancuso, Laboratorio di Virologia, UOC Microbologia, AOU “G. Martino ”, Messina; Teresa Pollicino, Laboratorio di Diagnostica Molecolare, UOSD Gestione Centralizzata dei Laboratori, AOU “G. Martino ”, Messina; Francesca Di Gaudio, Centro Regionale per la Qualità (CRQ), Palermo; Stefano Vullo, Stefano Reale, Istituto Zooprofilattico Sperimentale della Sicilia, Palermo; Maria Grazia Cusi, UOC Microbiologia e Virologia, Azienda Ospedaliera Universitaria Senese, Dipartimento di Biotecnologie Mediche, Università degli Studi di Siena, Siena; Gian Maria Rossolini, SOD Microbiologia e Virologia, AOU “Careggi”, Firenze; Mauro Pistello, UOC Virologia, AOU Pisana, Pisa; Antonella Mencacci, Barbara Camilloni, Microbiology and Clinical Microbiology, Department of Medicine and Surgery, University of Perugia, Perugia; Silvano Severini, Istituto Zooprofilattico Sperimentale dell’Umbria e delle Marche, Perugia; Massimo Di Benedetto, Laboratorio Analisi Cliniche, Ospedale “Parini”, Aosta; Calogero Terregino, Isabella Monne, Istituto Zooprofilattico Sperimentale delle Venezie, Legnaro, Padova; Valeria Biscaro, UOC Microbiologia-Virologia, AULSS2 La Marca, PO Treviso, Treviso.

## Supplementary Data

2849000 Supplement
