# Supplementary material for: Co-circulation of SARS-CoV-2 Alpha and Gamma variants in Italy, February and March 2021
Source: Euro Surveill. 2022 Feb 3;27(5):2100429. doi: 10.2807/1560-7917.ES.2022.27.5.2100429 (PMC8815098; doi:10.2807/1560-7917.ES.2022.27.5.2100429)
Supplement: Supplement [file 21-00429_STEFANELLI_Supplement.pdf]

This supplementary material is hosted by Eurosurveillance as supporting information alongside the article" Co-circulation of SARS-CoV-2 Alpha and Gamma variants in Italy, February-March 2021", on behalf of the authors, who remain responsible for the accuracy and appropriateness of the content. The same standards for ethics, copyright, attributions and permissions as for the article apply. Supplements are not edited by Eurosurveillance and the journal is not responsible for the maintenance of any links or email addresses provided therein

Supplementary Material

Contents

Alpha variant detection by RealTime RT-PCR........2
Three-strain SARS-CoV-2 model........2
Restrictions in the different tiers since January 14, 2021........7
Sensitivity analyses........10
Transmissibility of Alpha in regions without Gamma circulation........10
Increased duration of infection........12
Co-circulation of Alpha and Gamma in individual regions of the Central macro-area........13
Degree of cross-protection as a free parameter........16
Alternative assumptions on hospitalization probability........17
Co-circulation of historical lineages and Gamma in absence of Alpha........18
Abbreviations of regions........21

Alpha variant detection by RealTime RT-PCR

The primers were designed to include the nucleotide deletion at amino acid positions H69-V70 (sense primer), and Y144 (antisense primer) in the Spike Gene, in order to amplify only alpha variant sequences. Sense primer (19mer, nt 188-212): 5'_CTTGGTTCCATGCTATCTC. Antisense primer (22mer, nt 452-422 of the spike ORF of the original Wuhan sequence, acc. n° NC_045512): 5'TTTTGTTGTTTTTGTGGTAAAC. The probe was marked by the 5Cy5/TAO combination (22mer, nt 291-312): GTCTAACATAATAAGAGGCTGG. The amplification reaction was performed using the SuperScript III Platinum One-Step Quantitative RT-PCR System (Invitrogen by ThermoFisher), in a final volume of 25 µl containing 5 µl of sample RNA, 12,5 pmol of each primer and 100nM probe. The amplification profile was the following: stage 1: 55.0 °C, 1'; stage 2: 54.0°C, 3'; stage 3: 45 repetition of step 1: 94.0°C, 15'' and step 2: 62.0°C, 40''. Reactions were performed in a 7500 fast thermal cycler (Applied Biosystems).

Three-strain SARS-CoV-2 model

We adopted a three-strain Susceptible – Infectious – Recovered (SIR) mathematical model to simulate co-circulation of historical lineages of SARS-CoV-2 (“wildtype”) and variants of concern Alpha and Gamma.

The model is described by the following equations

$$\begin{cases} S'(t) = -\left(\lambda_{wt}(t) + \lambda_{v1}(t) + \lambda_{v2}(t)\right)S(t) \\ I'_{wt}(t) = \lambda_{wt}(t)S(t) - \gamma I_{wt}(t) \\ I'_{v1}(t) = \lambda_{v1}(t)S(t) - \gamma I_{v1}(t) \\ I'_{v2}(t) = \lambda_{v2}(t)S(t) + (1-\alpha)\lambda_{v2}(t)(Z_{wt}(t) + Z_{v1}(t)) - \gamma I_{v2}(t) \\ Z'_{wt}(t) = \gamma I_{wt}(t) - (1-\alpha)\lambda_{v2}(t)Z_{wt}(t) \\ Z'_{v1}(t) = \gamma I_{v1}(t) - (1-\alpha)\lambda_{v2}(t)Z_{v1}(t) \\ Z'_{v2}(t) = \gamma I_{v2}(t) \end{cases}$$

where:

- S represents individuals never infected with SARS-CoV-2 infection of any lineage;
- wt, v1 and v2 are subscripts for historical lineages, Alpha and Gamma, respectively;
- I_x represents individuals infected with lineage x;
- Z_x represent individuals who have recovered from infection with lineage x;
- $\lambda_x = \beta_x \frac{I_x}{N}$ is the force of infection of lineage x, where β_x is the transmission rate of lineage x and $N = S + \sum_x(I_x + Z_x)$ is the total population; we assume $\beta_{v1} = k_1\beta_{wt}$ and $\beta_{v2} = k_2\beta_{wt}$, where k_1 and k_2 are the relative transmissibilities of Alpha and Gamma with respect to the historical lineages;
- α is the degree of cross-protection granted from previous infection with historical or Alpha lineages against re-infection with Gamma (α=1 is complete cross-protection); we assumed that recovering from any infection provides full immunity against re-infection with historical or Alpha lineages for at least the duration of our simulations (3 months).
- γ is the recovery rate from infection, set equal to the inverse of the average generation time previously estimated for Italy at 6.6 days [1].

The initial fraction of immune individuals was computed as

$$Z_0 = \frac{\sum_{T=1,2,3} \frac{C_T}{\chi_T}}{N}$$

Where T is one of three time periods (1: February 20 – June 30, 2020, 2: July 1 – September 30, 2020; 3: October 1, 2020 – January 15, 2021), C_T is the cumulative number of cases notified in period T, χ_T is the infection notification rate in the period (estimated at χ_1=9.4%, χ_2=24.5% and χ_3= 30% [2]). We approximated all initially immune individuals to be due to the wildtype lineage.

Table S1. Model values by geographical aggregation.

PARAMETER	EXPRESSION	ITALY *	CENTER *	NORTH-EAST	SOUTH
Population (millions)	N	58.7	10.5	11.7	13.9
Fraction initially immune (%)	Z_0	16.0%	12.4%	21.1%	10.3%

***** Excluding Marche

The reproduction number of historical lineages on January 15, 2021 was computed as:

$$R_{wt} = \frac{\beta_{wt}}{\gamma}(1 - Z_0)$$

The number of infectious individuals I_0 from any lineage was initialized by sampling from a Poisson distribution with rate equal to the mean number of daily hospital admissions between January 10 and January 17, multiplied by the average generation time and dividing by the average proportion *h* of hospitalized infections who require hospitalization, estimated by applying the method reported in [2]. Previous estimates on this parameter reported an approximately 13% hospitalization probability for symptomatic cases admitted between March and September 2020 [2]. When re-applying the same methodology to cases from October 2020 to January 15, we obtained a 7.7% hospitalization probability among symptomatic individuals. Taking into account the overall proportion of infections who become symptomatic [3], we estimate that 2.4% of all infections required hospitalization between October 2020 and January 2021. However, we run sensitivity analyses with alternative values of *h* of 1.2% and 3.6% (see below).
The initial number of infected individuals was distributed in the three compartments I_{v1}, I_{v2} and I_{wt}, according to the initial prevalence of the three lineages, f_1 , f_2, and $(1 - f_1 - f_2)$, respectively.

The reproduction number of historical lineages on January 15, 2021, R_{wt}, the relative transmissibility of Alpha and Gamma, k_1 and k_2, the initial prevalence of Alpha and Gamma, f_1 and f_2, and the initial number of infectious individuals of any lineage I_0 were free model parameters. The degree of cross-protection α was fixed to values between 0 and 1 with a step of 0.1, and for each value of α the free model parameters were recalibrated. The model-estimated daily hospital admissions from any lineage were computed as the daily new infections, multiplied by the hospital admission probability and shifted forward by 3 days to allow for delays between the start of infectiousness and the hospital admission. The model-estimated prevalence of lineage x at time t, used for the multinomial likelihood (see main text), was computed as $p_t^x = \frac{I_x(t)}{\sum_q I_q(t)}$ where q={wt, v1, v2}.

For calibration via MCMC, we used uninformative priors with 50,000 iterations (of which the first 10,000 were discarded to consider only iterations after convergence) and a Manhattan algorithm with recursive normal jumps for accepting or rejecting candidate parameter sets. Because the definition of the likelihood includes only relative prevalence data (see main text), we constrained the MCMC to explored parameter values for which the model-estimated absolute incidence was close to realistic values; to this aim, we assigned L=0 to simulations for which the mean square error between the modeled and observed daily hospital admissions (a reliable proxy of the total incidence) is below 1.5 times the variance of the observations. Convergence was assessed via visual inspection of the MCMC traceplots. The

overall code, including the MCMC algorithm was programmed in R version 4.0.3 (2020-10-10).

Figure S1 shows posterior means and 95% credible intervals (CrI) for the estimated initial prevalence of Alpha and Gamma, f_1 and f_2, in Italy and in the three macro-areas where, according to the surveys, Alpha and Gamma are co-circulating. These estimates are insensitive to different values of α.

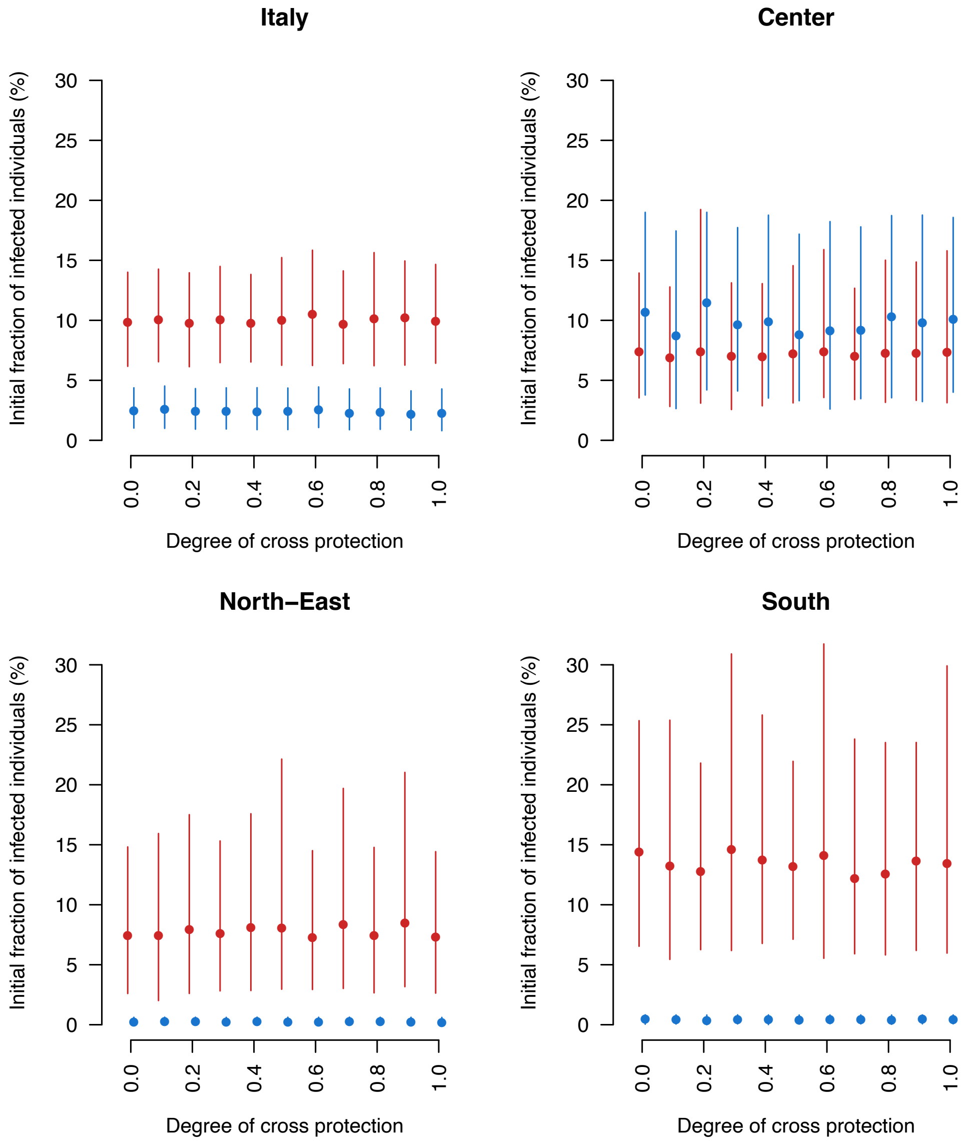

Figure S1. Posterior distribution of the initial prevalence of Alpha (in red) and Gamma (in blue) across different geographical aggregations. Dots represent posterior mean values and lines indicate 95% CrI.

Figure S2 shows the posterior means and 95% credible intervals for estimated reproduction number of historical lineages on January 15, R_{wt}. These estimates are insensitive to different values of α as well.

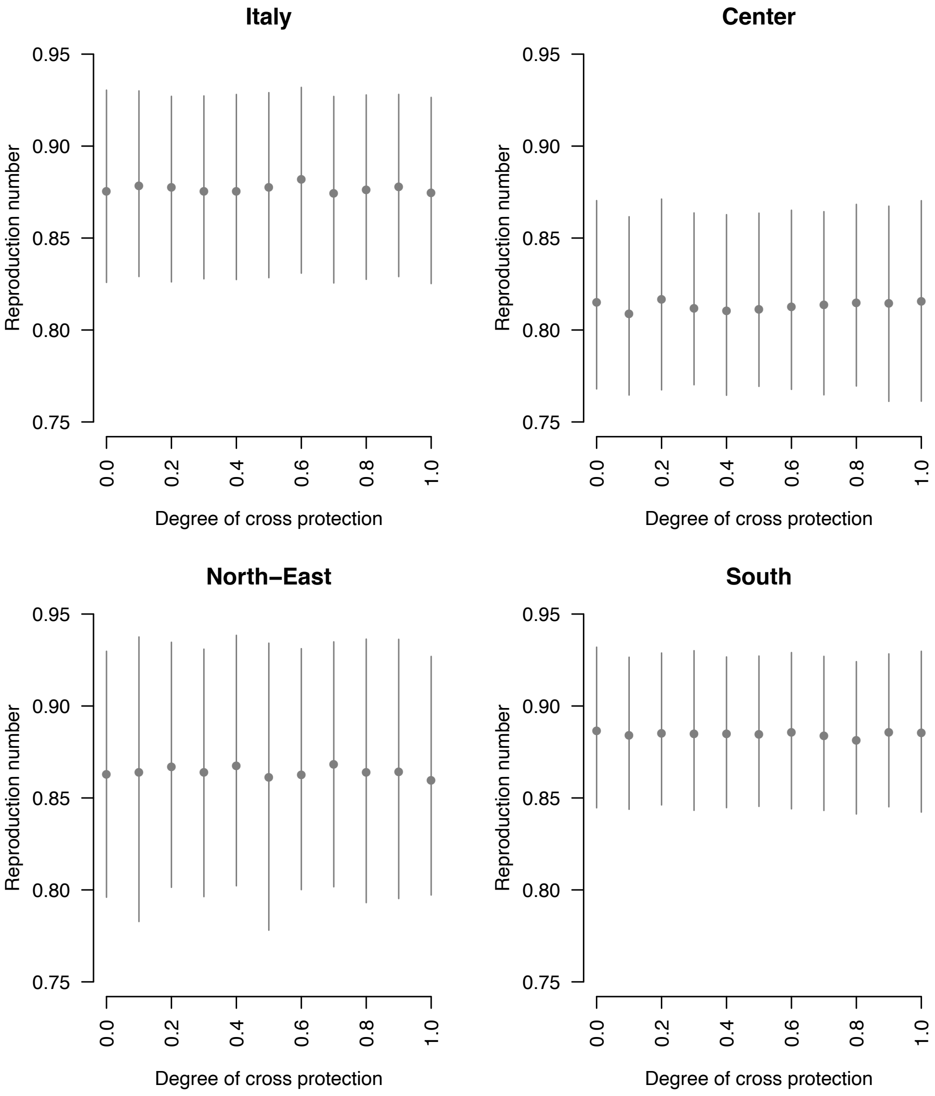

Figure S2. Posterior distribution of the reproduction number of historical lineages on January 15, 2021, R_{wt}, across different geographical aggregations. Dots represent posterior mean values and lines indicate 95% CrI.

Figure S3 shows the posterior means and 95% credible intervals for the initial number of infectious individuals of any lineage I_0. These estimates are insensitive to different values of α as well.

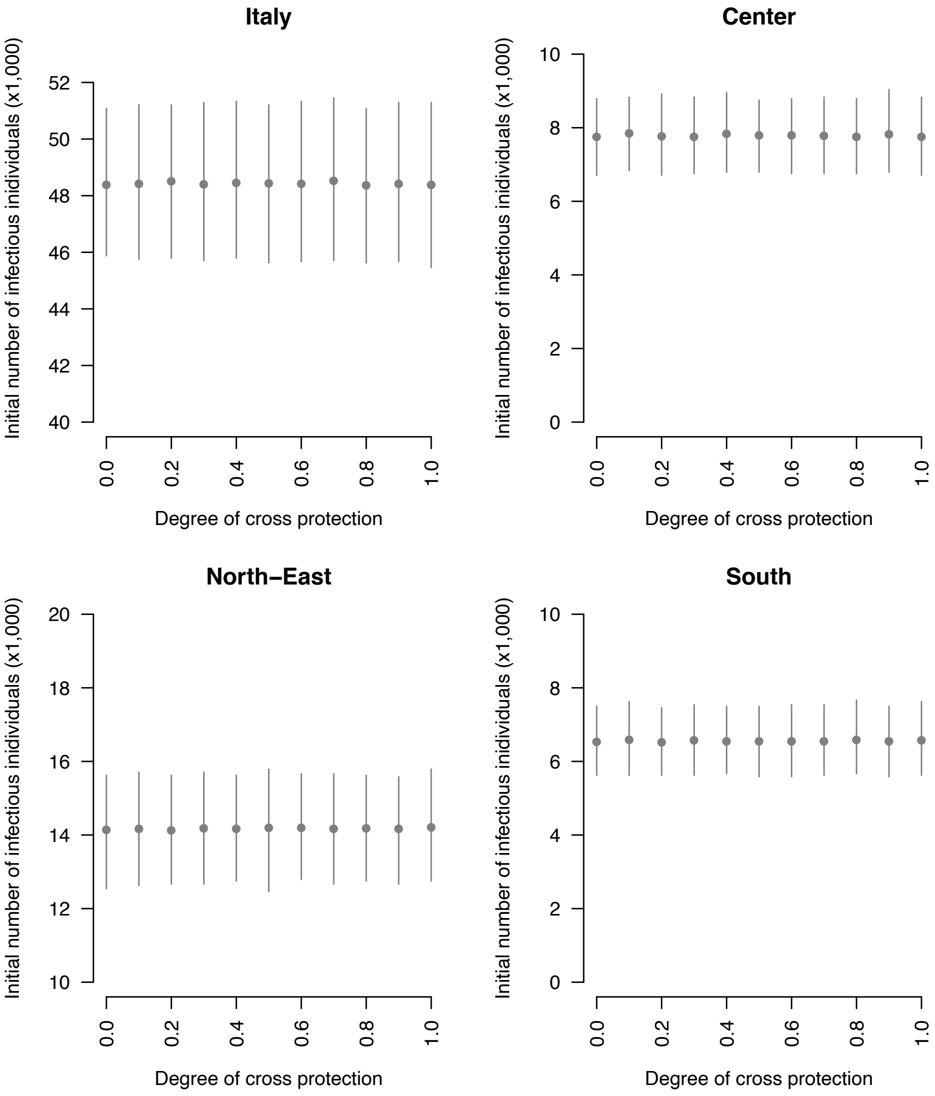

Figure S3. Posterior distribution of the initial number of infectious individuals of any lineage $\boldsymbol{I_0}$, across different geographical aggregations. Dots represent posterior mean values and lines indicate 95% CrI.

Restrictions in the different tiers since January 14, 2021

A restriction system based on tiers was introduced in Italy on a regional basis since November 6, 2021. Table S2 and S3 show restrictions associated to each tier adopted between January 14, 2021 and March 1, 2021, and between March 2, 2021 and April 2, 2021.
Since March 2, 2021, as shown in TabS3, a new tier ("white") was introduced for regions and autonomous provinces where all quantitative indicators indicate a low risk of SARS-CoV2 transmission. Moreover, restrictions were made stricter in orange and red tiers, especially those relative to the educational system

Table S2. Description of restrictions applied in Italy since January 14, 2021.

**Restrictions**	**January 14, 2021 https://www.gazzettaufficiale.it/eli/id/2021/01/15/21A00221/sg**		
	Yellow tier	Orange tier	Red tier
**Face masks**	Mandatory in outdoor spaces	Mandatory in outdoor spaces	Mandatory in outdoor spaces
**Individual movements**	Stay-home mandate between 10pm and 5am, no movements between region (except for work, health and other certified reasons).	Stay-home mandate between 10pm and 5am and ban on movements between municipalities and to/from other regions (except for work, health and other certified reasons)	Stay-home mandate and ban on movements between municipalities and to/from other regions (except for work, health and other certified reasons).
**Retail and Services**	Open with capacity reduction	Open with capacity reduction	All shops closed (with the exception of essential retail & services)
**Schools & Childcare**	Recommendation to adopt distance learning for universities Guaranteed in-presence learning for at least 50% and up to 75% of the time in high schools. in-presence for lower educational institution. Mandatory personal protection devices	Recommendation to adopt distance learning for universities Guaranteed in-presence learning for at least 50% and up to 75% of the time in high schools. in-presence for lower educational institution. Mandatory personal protection devices	Distance learning in second and third grade of middle schools, in all grades of high schools and universities
**Bars serving food, Cafès & Restaurants**	Open with capacity reduction Only take away allowed after 6pm until 10pm. Closed from 10pm to 5am.	Closed from 10pm to 5am. Take away allowed until 10pm.	Closed from 10pm to 5am. Take away allowed until 10pm.
**Public transport**	50% capacity reduction	50% capacity reduction (except school service)	50% capacity reduction (except school service)
**Indoor recreational and cultural venues**	Closed	Closed	Closed
**Gyms, pools & leisure venues**	Non-professional sports and professional contact sports not permitted. Closed except outdoor sport centers; ski centers are closed (except sport events of national interest)	Closed except outdoor sport centers; ski centers are closed (except sport events of national interest)	Closed. Individual outdoor training only (except sport events of national interest)

Table S3. Description of restrictions applied in Italy since March 2, 2021.

**Restrictions**	**March 2, 2021 – 2 April 2021 https://www.gazzettaufficiale.it/eli/id/2021/03/02/21A01331/sg**			
	White tier	Yellow tier	Orange tier	Red tier
**Face masks**	Mandatory in outdoor spaces	Mandatory in outdoor spaces	Mandatory in outdoor spaces	Mandatory in outdoor spaces
**Individual movements**	No gatherings	Stay-home mandate between 10pm and 5am (except for work, health and other certified reasons).	Stay-home mandate between 10pm and 5am and ban on movements between municipalities and to/from other regions (except for work, health and other certified reasons)	Stay-home mandate and ban on movements between municipalities and to/from other regions (except for work, health and other certified reasons).
**Retail and Services**	Open	Shopping malls closed during weekends and holidays (with the exception of essential retail & services)	Shopping malls closed during weekends and holidays (with the exception of essential retail & services)	All shops closed (with the exception of essential retail & services)
**Schools & Childcare**	Open	Recommendation to adopt distance learning for universities Guaranteed in-presence learning for at least 50% and up to 75% of the time in high schools. in-presence for lower educational institution. Mandatory personal protection devices	Distance learning in high schools and universities except when on-site attendance is essential (i.e., for laboratory activities)	Distance learning in all grades of high schools and universities. Closed childcare
**Bars serving food, Cafès & Restaurants**	Open with personal protection devices	Open with capacity reduction from 5am to 6pm. No service after 6pm and take away allowed until 10pm.	Closed. Home delivery and and take away allowed until 10pm allowed	Closed from 10pm to 5am. Take away and Home delivery allowed until 10pm
**Public transport**	Open with personal protection devices	50% capacity reduction (except school service)	50% capacity reduction (except school service)	50% capacity reduction (except school service)
**Indoor recreational and cultural venues**	Open with personal protection devices and capacity reduction	Outdoors gatherings allowed with social distancing measures. Indoors with capacity reduction, social distancing and personal protection measures	Closed	Closed
**Gyms, pools & leisure venues**	Open with personal protection devices and capacity reduction	Closed except outdoor sport centers; ski centers are closed (except sport events of national interest)	Closed except outdoor sport centers; ski centers are closed (except sport events of national interest)	Closed. Individual outdoor training only (except sport events of national interest)

Sensitivity analyses

Transmissibility of Alpha in regions without Gamma circulation

We conducted a sensitivity analysis to estimate the transmissibility of Alpha in Lombardy and Veneto, in which Gamma was not circulating and for which an additional data point from an identical survey conducted on cases diagnosed on February 3 and 4 was available (Table S4).

Table S4. Results of the survey on cases diagnosed on February 3-4, 2021 In Lombardy and Veneto regions. 'RT-PCR positive' represent only cases tested in the labs involved and not all positive cases in a region.

REGION	LABS	RT-PCR POSITIVE	SEQUENCED SAMPLES	ANALYZED SAMPLES	SAMPLES POSITIVE to Alpha	POINT PREVALENCE OF Alpha (95% CI)
LOMBARDY	7	229	229	229	79	34.5% (28.4-41.0)
VENETO	10	182	182	164	29	17.7% (12.2-24.4)

The estimate was conducted using a two-strain model that results from a special case of the three-strain model when parameter f_2 is fixed to zero.

Table S5. Initial values in the model.

PARAMETER	EXPRESSION	LOMBARDY	VENETO
Population (millions)	N	10.1	4.9
Fraction initially immune (%)	Z_0	23.6%	22.9%

As shown in Figure S4, the model was able to fit the epidemiological trends on hospital admissions and the prevalence of Alpha estimated in the three surveys.

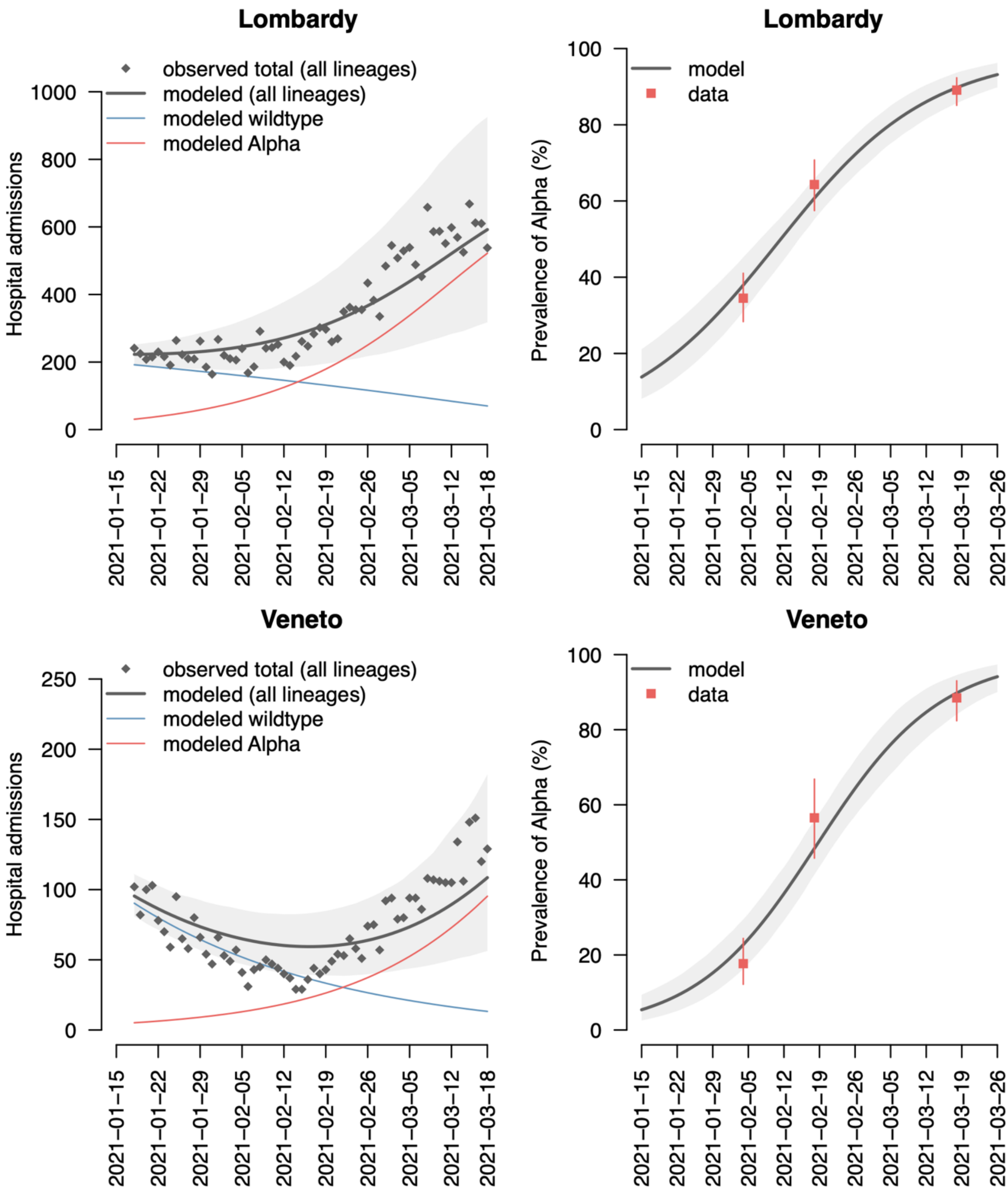

Figure S4. Model fits. Left column: model-estimated and observed hospital admissions over time for Lombardy (top panel) and Veneto (bottom panel). Black lines represent mean values of model-estimated overall daily hospitalizations, shaded areas indicate 95%CrI. Colored lines indicate mean values of model-estimated daily hospitalizations attributable to wildtype (blue) and Alpha (red) lineages. Black points indicate observed daily hospitalizations. Right column: model-estimated (solid black lines: mean values; shaded areas: 95% CrI) and observed (red points: mean values; red lines: 95%CI) prevalence of Alpha in Lombardy (top panel) and Veneto (bottom panel). The model-estimated prevalence is computed as the ratio at each time of the modeled number of currently infectious individuals with lineage Alpha and the modeled total number of currently infectious individuals with any lineage.

The estimated relative transmissibility of Alpha was 1.49 (95%CI: 1.36-1.66) in Lombardy and 1.72 (95%CI: 1.52-1.98) in Veneto, in line with those obtained for other aggregations and in presence of co-circulation with Gamma in the main analysis.

Increased duration of infection

We run a sensitivity analysis for Lombardy and Veneto regions where instead of considering the generation time equal for historical and Alpha lineages, we leave it as a free model parameter, while we assumed that the transmissibility of the two lineages were the same. As shown in Figure S5, the model was able to fit the epidemiological trends on hospital admissions and the prevalence of Alpha estimated in the three surveys.

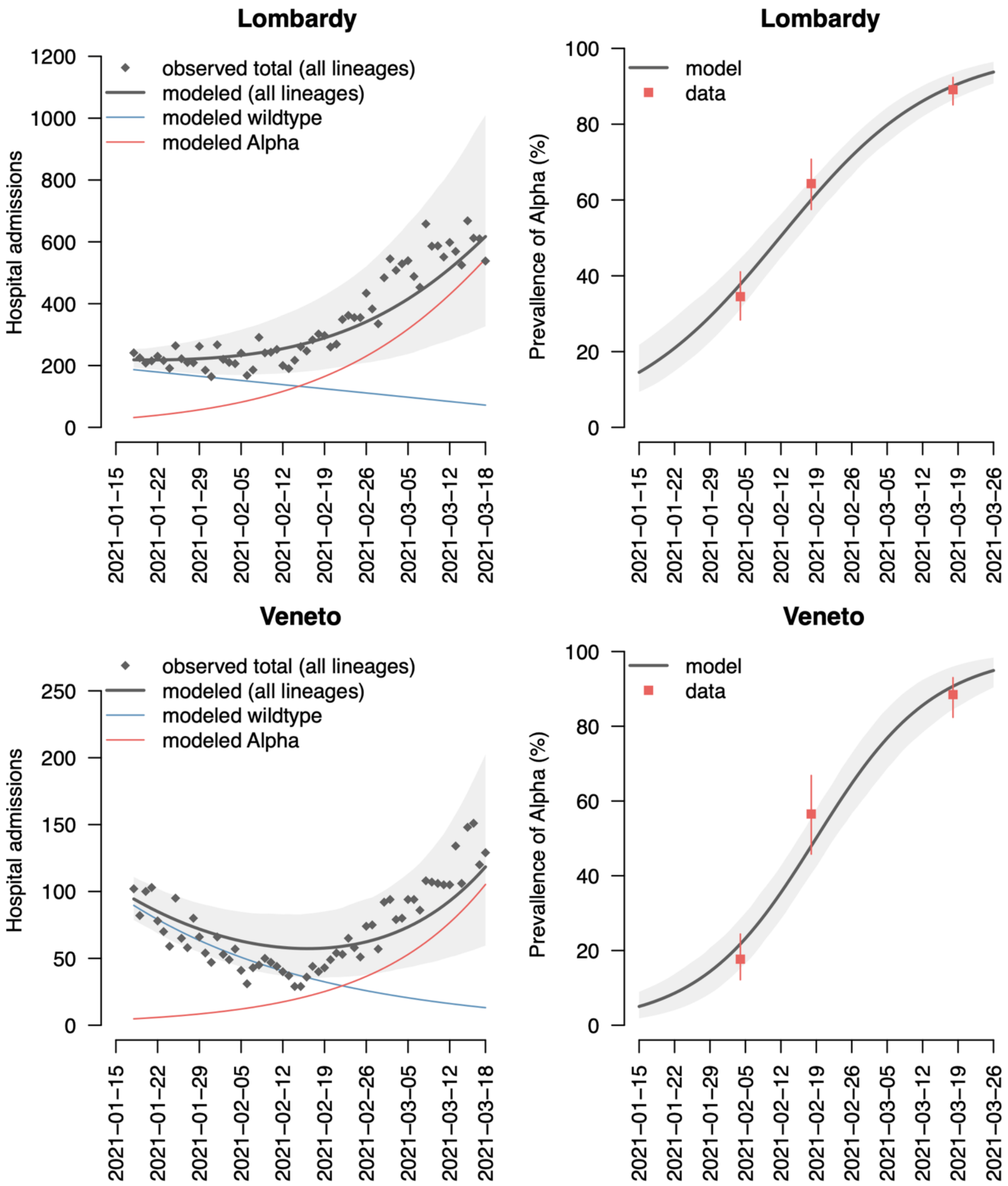

Figure S5. Model fits. Left column: model-estimated and observed hospital admissions over time for Lombardy (top panel) and Veneto (bottom panel). Black lines represent mean values of model-estimated overall daily hospitalizations, shaded areas indicate 95%CrI. Colored lines indicate mean values of model-estimated daily

hospitalizations attributable to wildtype (blue) and Alpha (red) lineages. Black points indicate observed daily hospitalizations. Right column: model-estimated (solid black lines: mean values; shaded areas: 95% CrI) and observed (red points: mean values; red lines: 95%CI) prevalence of Alpha in Lombardy (top panel) and Veneto (bottom panel). The model-estimated prevalence is computed as the ratio at each time of the modeled number of currently infectious individuals with lineage Alpha and the modeled total number of currently infectious individuals with any lineage.

The estimated relative duration of infection of Alpha was 1.79 (95%CI: 1.50-2.18) in Lombardy and 2.52 (95%CI: 1.74-4.56) in Veneto. These estimates translate into an average duration of infection for Alpha lineage of 11.8 days (95%CI: 9.9-14.4) for Lombardy and 16.6 days (95%CI: 11.5-30.1) for Veneto.

Co-circulation of Alpha and Gamma in individual regions of the Central macro-area

We conducted a sensitivity analysis in two individual regions of the Central macro-area with high co-circulation (Tuscany and Lazio) to investigate the potential impact of geographical aggregation on estimates of the relative transmissibility of Alpha and Gamma.

Table S6. Initial values in the model.

PARAMETER	EXPRESSION	TUSCANY	LAZIO
Population (millions)	N	3.7	5.9
Fraction initially immune (%)	Z_0	13.4%	11.7%

As shown in Figures S6 and S7, the model was able to fit the epidemiological trends on hospital admissions and the prevalence of Alpha and Gamma estimated in the two surveys in both regions.
As shown in Figure S8, independently on the assumed degree of cross-protections, we found a robust mean estimate for the relative transmissibility of Alpha for Lazio, ranging between 1.80 and 1.85, with confidence intervals ranging between 1.68 and 2.06, and for Tuscany, ranging between 1.54 and 1.69, with confidence intervals ranging between 1.23 and 2.17, compatible with estimates from the main analysis.

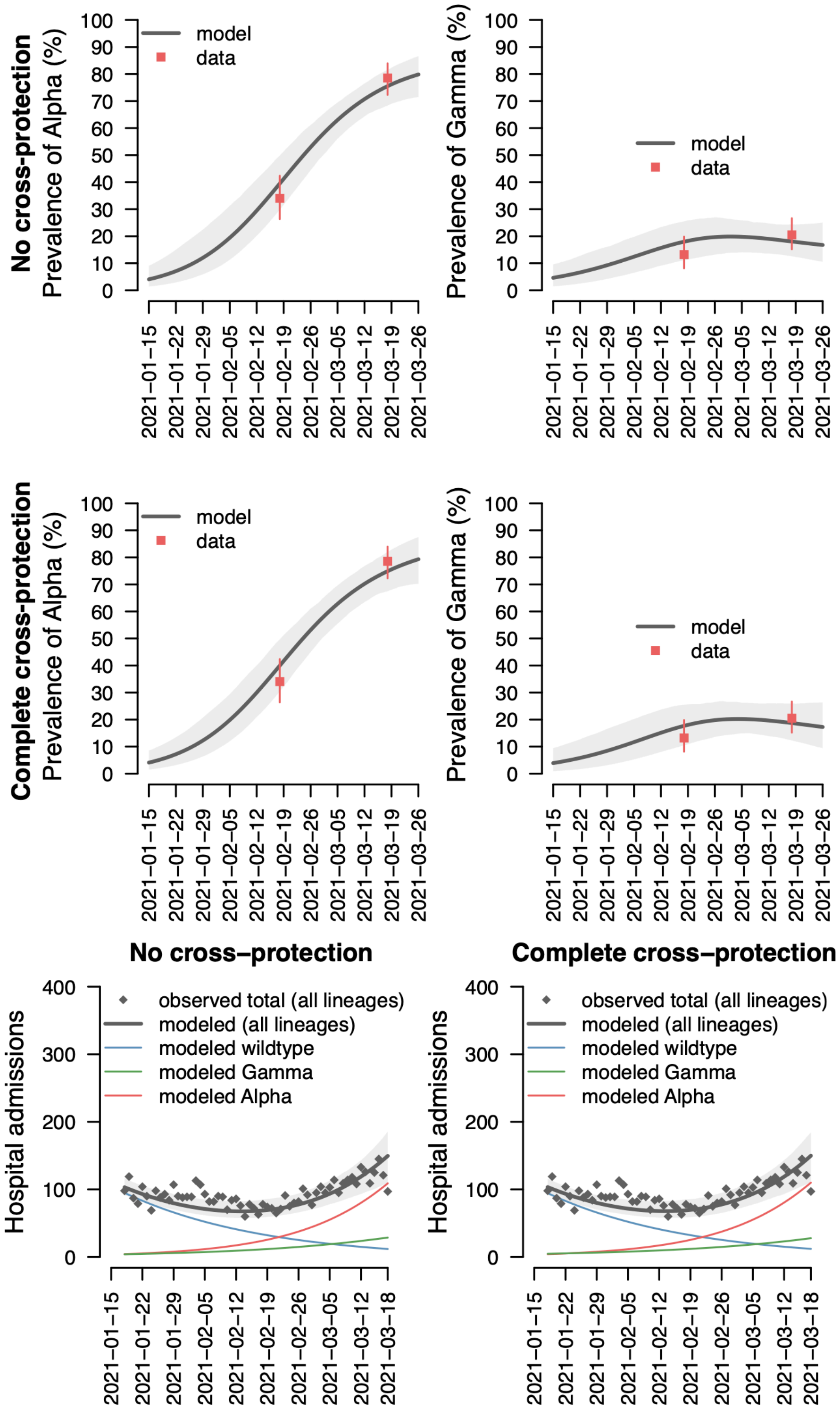

Figure S6. Model fits for Lazio. Top row: model-estimated (solid black lines: mean values; shaded areas: 95% CrI) and observed (red points: mean values; red lines: 95%CI) prevalence of Alpha (left) and Gamma (right) when assuming no cross-protection between wildtype or Alpha and Gamma. Middle row: as top row but assuming complete cross-protection among all lineages. The model-estimated prevalence is computed as the ratio at each time of the modeled number of currently infectious individuals with lineage Alpha and the modeled total number of currently infectious individuals with any lineage. Bottom row: model-estimated and observed hospital admissions over time. Black lines represent mean values of model-estimated overall daily hospitalizations, shaded areas indicate 95%CI. Colored lines indicate mean values of model-estimated daily

hospitalizations attributable to wildtype (blue), Alpha (red) and Gamma (green) lineages. Black points indicate observed daily hospitalizations.

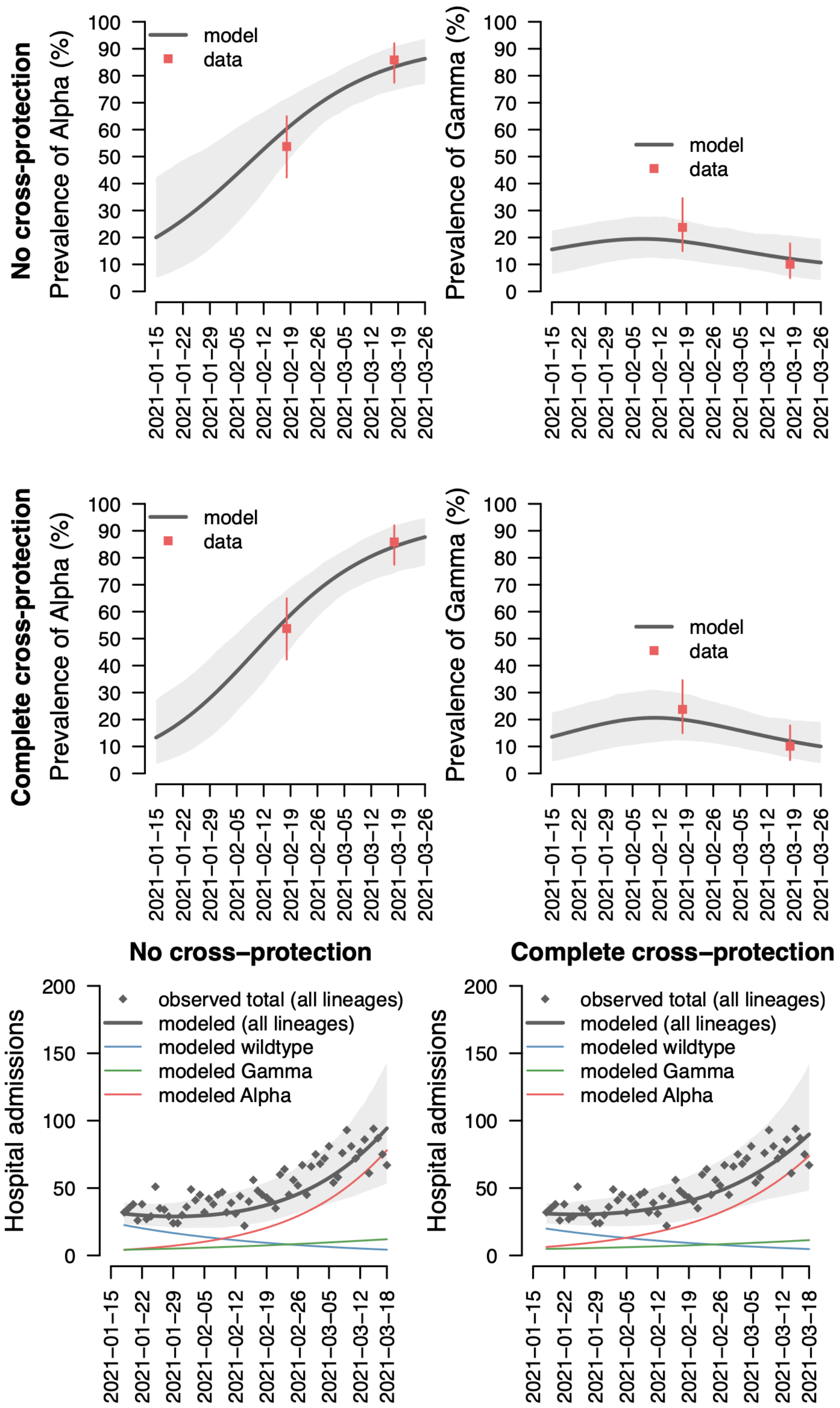

Figure S7. Model fits for Tuscany. Top row: model-estimated (solid black lines: mean values; shaded areas: 95% CrI) and observed (red points: mean values; red lines: 95%CI) prevalence of Alpha (left) and Gamma (right) when assuming no cross-protection between wildtype or Alpha and Gamma. Middle row: as top row but assuming complete cross-protection among all lineages. The model-estimated prevalence is computed as the ratio at each time of the modeled number of currently infectious individuals with lineage Alpha and the modeled total number of currently infectious individuals with any lineage. Bottom row: model-estimated and observed

hospital admissions over time. Black lines represent mean values of model-estimated overall daily hospitalizations, shaded areas indicate 95%CI. Colored lines indicate mean values of model-estimated daily hospitalizations attributable to wildtype (blue), Alpha (red) and Gamma (green) lineages. Black points indicate observed daily hospitalizations.

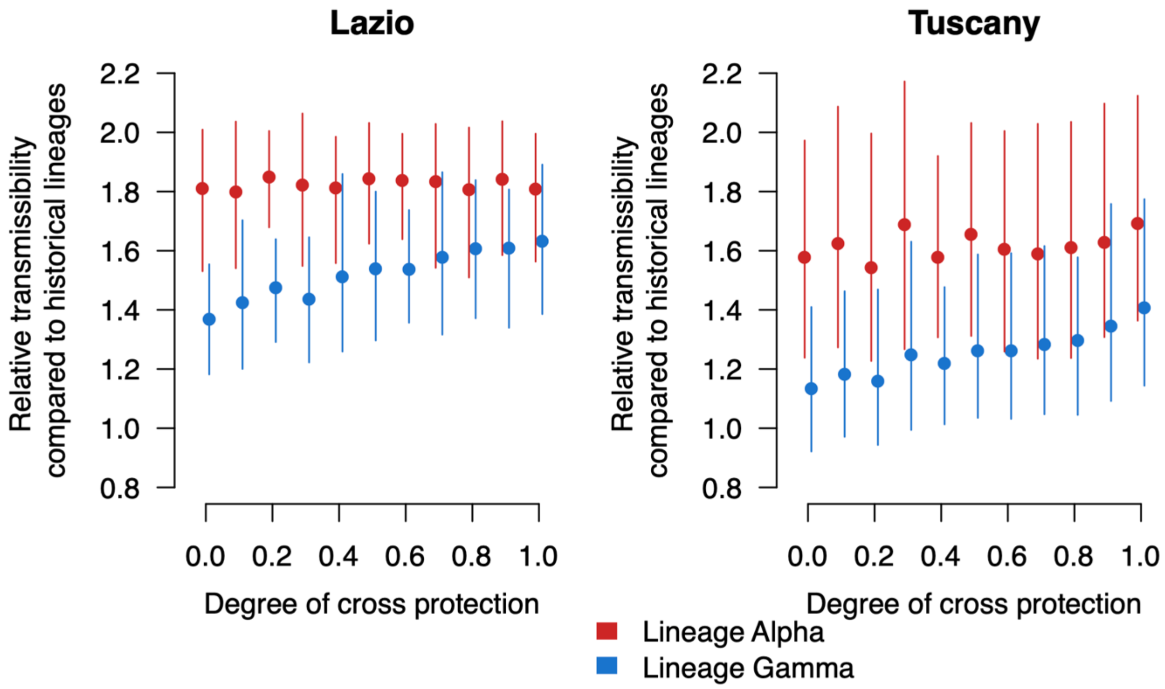

Figure S8. Estimates of the relative transmissibility of Alpha and Gamma. Estimates are provided for different assumed values on the degree of cross protection (0: no cross-protection; 1: complete cross-protection) conferred by previous infection with the wildtype or Alpha against reinfection with Gamma. Points indicate the mean value of the estimated relative transmissibility of Alpha (red) and Gamma (blue) lineages; lines indicate 95%CI.

Degree of cross-protection as a free parameter

We run a sensitivity analysis for Italy where instead of assuming the degree of cross-protection between historical/Alpha lineages and Gamma, we estimate it as a free model parameter. As shown in Figure S9, the estimated degree of cross-protection may take values between 0 and 1 and it has a strong positive correlation with the estimated relative transmissibility of Gamma (correlation = 0.78, p-value < 0.001). The estimated relative transmissibility of Alpha and Gamma at national level are respectively 1.56 (95%CI: 1.48-1.65) and 1.24 (95%CI: 1.01-1.44), consistently with the main analysis.

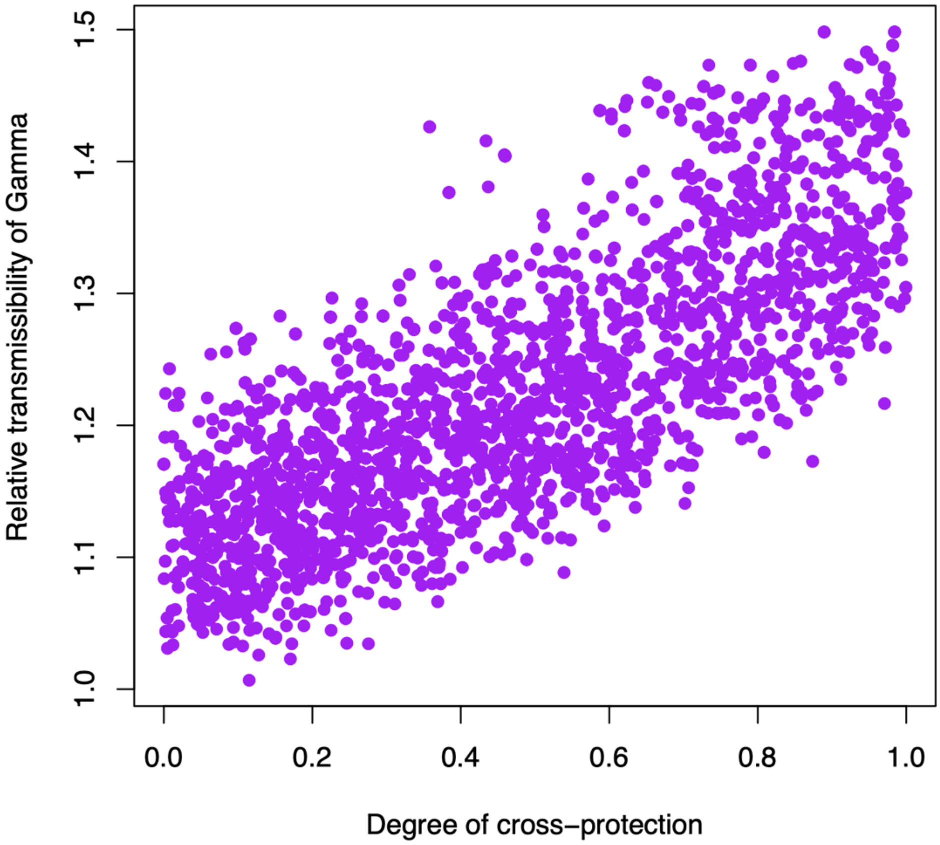

Figure S9. Posterior estimates. Scatterplot of joint posterior MCMC estimates of the relative transmissibility of Gamma and with degree of cross-protection.

Alternative assumptions on hospitalization probability

We run sensitivity analyses for Italy where we made different assumptions on the hospitalization probability *h*. In particular, we assumed i) a 50% lower value (*h*=1.2%), applied to all lineages; ii) a 50% higher value (*h*=3.6%), and iii) a 50% increased value in the hospitalization probabilities of Alpha and Gamma lineages only, to account for a possible increased severity [4, 5].
In all three analyses, the model was able to fit the epidemiological trends on hospital admissions and the prevalence of Alpha and Gamma estimated in the two surveys in Italy.

As shown in Figure S10, under the three different assumptions on the hospitalization probabilities, we found robust mean estimates for the relative transmissibility of Alpha and Gamma, compatible with estimates from the main analysis.

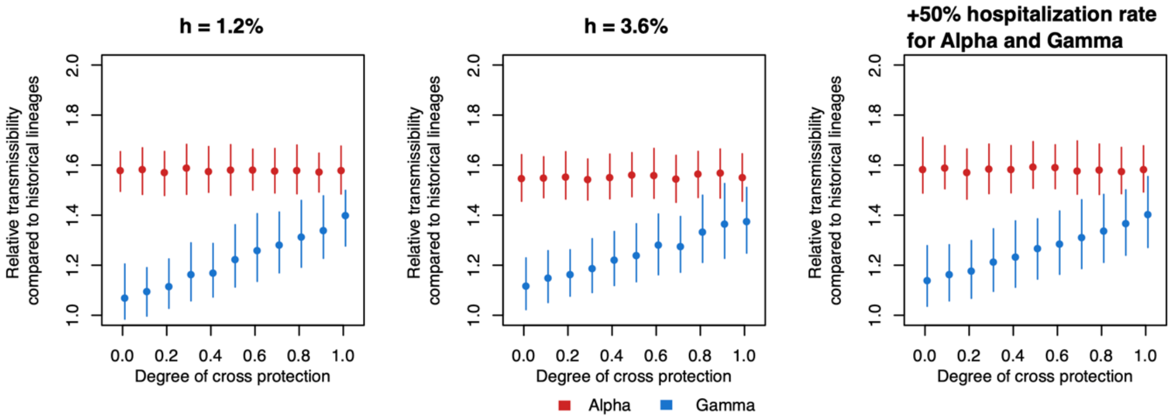

Figure S10. Estimates of the relative transmissibility of Alpha and Gamma. Estimates are provided for different assumed values on the degree of cross protection (0: no cross-protection; 1: complete cross-protection) conferred by previous infection with the wildtype or Alpha against reinfection with Gamma, under the assumption of a 50% lower hospitalization probability (left), a 50% higher hospitalization probability (middle), and a 50% increase in the hospitalization probability for Alpha and Gamma lineages only (right). Points indicate the mean value of the estimated relative transmissibility of Alpha (red) and Gamma (blue) lineages; lines indicate 95%CI.

Co-circulation of historical lineages and Gamma in absence of Alpha

We evaluated a theoretical scenario where we assume co-circulation of historical lineages and Gamma but in absence of Alpha. We implement this by sampling parameters from posterior distributions estimated in the baseline analysis and forcing the value of f_1 to zero. We computed these simulations for two geographical aggregations: the national level and the Center macro-area, where Gamma. was more prevalent. As shown in Figure S11, in absence of Alpha, the Gamma lineage would have reached dominance (>50% prevalence) in Italy by early April, independently on assumptions on the degree of cross-protection. In the Central macro-area, where the observed prevalence of Gamma during the two surveys was highest, the Gamma lineage would have reached dominance (>50% prevalence) by late February, independently on assumptions on the degree of cross-protection, as shown in Figure S12.

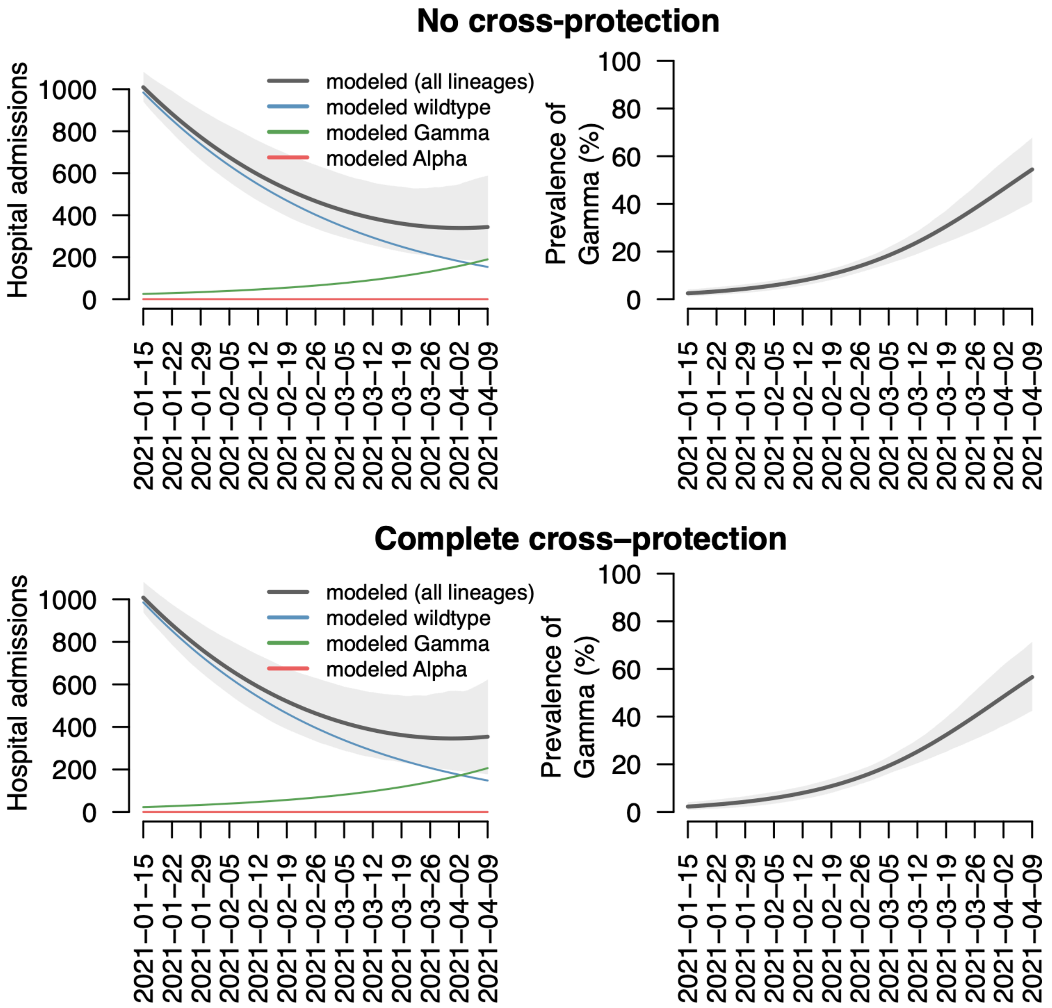

Figure S11. Model projections for Italy under a theoretical scenario with co-circulation of historical and Gamma lineages, in absence of Gamma. Left column: model-estimated hospital admissions over time when assuming no cross-protection (top) and complete cross-protection (bottom). Black lines represent mean values of model-estimated overall daily hospitalizations, shaded areas indicate 95%CI. Colored lines indicate mean values of model-estimated daily hospitalizations attributable to wildtype (blue), Alpha (red) and Gamma (green) lineages. Right column: model-estimated prevalence of Gamma when assuming no cross-protection (top) and complete cross-protection (bottom). Black lines represent mean values and shaded areas indicate 95%CI.

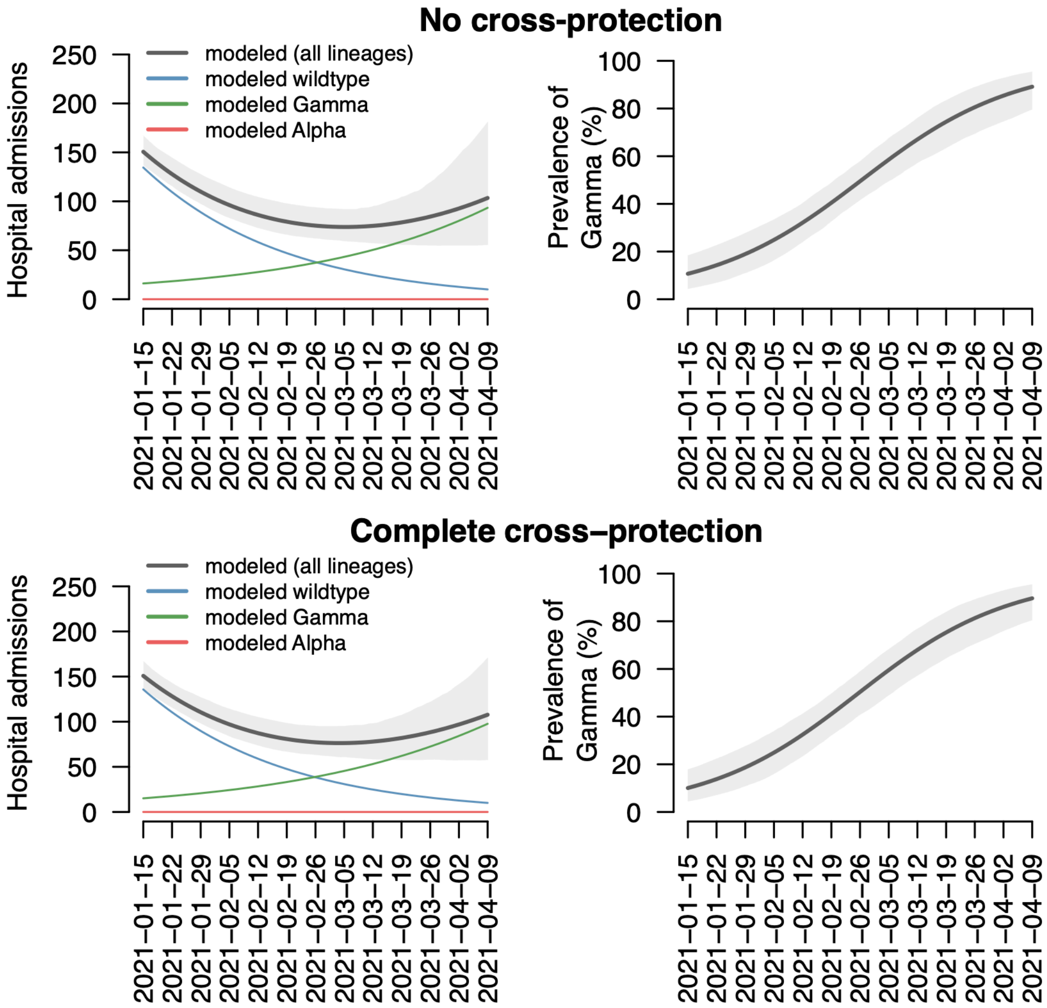

Figure S12. Model projections for the Central macro-area under a theoretical scenario with co-circulation of historical and Gamma lineages, in absence of Gamma. Left column: model-estimated hospital admissions over time when assuming no cross-protection (top) and complete cross-protection (bottom). Black lines represent mean values of model-estimated overall daily hospitalizations, shaded areas indicate 95%CI. Colored lines indicate mean values of model-estimated daily hospitalizations attributable to wildtype (blue), Alpha (red) and Gamma (green) lineages. Right column: model-estimated prevalence of Gamma when assuming no cross-protection (top) and complete cross-protection (bottom). Black lines represent mean values and shaded areas indicate 95%CI.

Abbreviations of regions

Table S7. Abbreviations of regions for Figure 2 in the main text.

Abbreviation	REGION
ABR	Abruzzo
AOV	Aosta Valley
APU	Apulia
BAS	Basilicata
BOL	Bolzano (AP)
CAL	Calabria
CAM	Campania
EMR	Emilia-Romagna
FVG	Friuli Venezia-Giulia
LAZ	Lazio
LIG	Liguria
LOM	Lombardy
MAR	Marche
MOL	Molise
PIE	Piedmont
SAR	Sardegna
SIC	Sicily
TRE	Trento (AP)
TUS	Tuscany
UMB	Umbria
VEN	Veneto

References

[1] Cereda D, *et al*. The early phase of the COVID-19 outbreak in Lombardy, Italy. 2020. *arXiv preprint* arXiv:2003.09320.
[2] Marziano V, *et al.* Retrospective analysis of the Italian exit strategy from COVID-19 lockdown. 2021. *Proc. Natl. Acad. Sci.* 118, e2019617118 (2021).
[3] Poletti, P., *et al*., 2021. Association of Age With Likelihood of Developing Symptoms and Critical Disease Among Close Contacts Exposed to Patients With Confirmed SARS-CoV-2 Infection in Italy. *JAMA network open*, *4*(3), pp.e211085-e211085.
[4] Davies NG, Jarvis CI, CMMID COVID-19 Working Group, Edmunds WJ, Jewell NP, Diaz-Ordaz K, Keogh RH. Increased mortality in community-tested cases of SARS-CoV-2 lineage ALPHA. Nature, 2021
[5] Challen R, Brooks-Pollock E, Read JM, Dyson L, Tsaneva-Atanasova K, Danon L. Risk of mortality in patients infected with SARS-CoV-2 variant of concern 202012/1: matched cohort study. BMJ 2021; 372:n579
